# A haustorial‐expressed lytic polysaccharide monooxygenase from the cucurbit powdery mildew pathogen *Podosphaera xanthii* contributes to the suppression of chitin‐triggered immunity

**DOI:** 10.1111/mpp.13045

**Published:** 2021-03-19

**Authors:** Álvaro Polonio, Dolores Fernández‐Ortuño, Antonio de Vicente, Alejandro Pérez‐García

**Affiliations:** ^1^ Departamento de Microbiología Facultad de Ciencias Universidad de Málaga Málaga Spain; ^2^ Instituto de Hortofruticultura Subtropical y Mediterránea ‘La Mayora’ Universidad de Málaga Consejo Superior de Investigaciones Científicas (IHSM−UMA−CSIC) Málaga Spain

**Keywords:** chitooligosaccharides, effectors, haustorium, LPMO, powdery mildews, RNAi silencing

## Abstract

*Podosphaera xanthii* is the main causal agent of cucurbit powdery mildew and a limiting factor of crop productivity. The lifestyle of this fungus is determined by the development of specialized parasitic structures inside epidermal cells, termed haustoria, that are responsible for the acquisition of nutrients and the release of effectors. A typical function of fungal effectors is the manipulation of host immunity, for example the suppression of pathogen‐associated molecular pattern (PAMP)‐triggered immunity (PTI). Chitin is a major component of fungal cell walls, and chitin oligosaccharides are well‐known PAMP elicitors. In this work, we examined the role of PHEC27213, the most highly expressed, haustorium‐specific effector candidate of *P. xanthii*. According to different computational predictions, the protein folding of PHEC27213 was similar to that of lytic polysaccharide monooxygenases (LPMOs) and included a conserved histidine brace; however, PHEC27213 had low sequence similarity with LPMO proteins and displayed a putative chitin‐binding domain that was different from the canonical carbohydrate‐binding module. Binding and enzymatic assays demonstrated that PHEC27213 was able to bind and catalyse colloidal chitin, as well as chitooligosaccharides, acting as an LPMO. Furthermore, RNAi silencing experiments showed the potential of this protein to prevent the activation of chitin‐triggered immunity. Moreover, proteins with similar features were found in other haustorium‐forming fungal pathogens. Our results suggest that this protein is a new fungal LPMO that catalyses chitooligosaccharides, thus contributing to the suppression of plant immunity during haustorium development. To our knowledge, this is the first mechanism identified in the haustorium to suppress chitin signalling.

## Graphical abstract

PxLPMO1 is a haustorial‐expressed lytic polysaccharide monooxygenase of *Podosphaera xanthii* that catalyses the transformation of immunogenic chitin fragments into small chitooligosaccharides, suppressing chitin signalling.

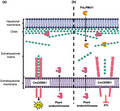

## 1 INTRODUCTION

*Podosphaera xanthii* is the main causal agent of cucurbit powdery mildew, a disease that causes important yield losses in cucurbit crops (Bellón‐Gómez et al., 2015; Fernández‐Ortuño et al., 2006; Pérez‐García et al., 2009; del Pino et al., 2002). Like all powdery mildew fungi, *P. xanthii* is dependent on living plant cells to complete its asexual life cycle (Martínez‐Cruz et al., 2014; Vogel & Somerville, 2002; Weßling et al., 2012). In this cycle, conidia transported by wind are deposited onto the leaf of a susceptible host plant. Subsequently, conidial adhesion, germination, and penetration are necessary steps for disease establishment (Spanu et al., 2010). After penetration, the fungus forms a specialized parasitic structure inside plant epidermal cells called the haustorium, which is responsible for the exchange of factors with the plant, such as the acquisition of nutrients (Bindschedler et al., 2009; Both et al., 2005; Martínez‐Cruz et al., 2014; Micali et al., 2011). However, to complete this cycle, the pathogen needs to avoid the action of plant defence elements, such as enzymes (van den Burg et al., 2007; Delaunois et al., 2014) and receptors, that recognize pathogen‐associated molecular patterns (PAMPs) (de Jonge et al., 2011; Tanaka et al., 2010), activating the so‐called PAMP‐triggered immunity (PTI) (Pieterse et al., 2009). For this reason, fungal pathogens have had to evolve and adapt to their hosts by developing several strategies to overcome plant defence responses. In this way, they counter with effectors, small proteins acquired during the coevolution of plant‐pathogenic fungi and their hosts (Pieterse et al., 2009), which, among other functions, prevent the recognition of PAMPs by plant receptors (Jones & Dangl, 2006; Lo Presti et al., 2015; Sánchez‐Vallet et al., 2013).

A major component of the fungal cell wall and a well‐known PAMP is chitin (de Jonge et al., 2010; Kombrink & Thomma, 2013; Pieterse et al., 2009; Tanaka et al., 2013), which is a long‐chain polymer of β‐1,4‐*N*‐acetylglucosamine, a derivative of glucose (de Jonge et al., 2010; Liu, Li et al., 2012; Wan et al., 2008; Young et al., 2005). Chitin provides structural rigidity to the fungal cell wall and acts as the first line of defence of pathogenic fungi against plant‐secreted enzymes, such as chitinases (Kombrink & Thomma, 2013; Wan et al., 2008). As a consequence of the enzymatic activity of plant chitinases, chitin oligosaccharides are released from the fungal cell wall and can be recognized by the plant receptor CERK1, a transmembrane LysM‐containing receptor with an intracellular kinase domain, thereby promoting chitin‐specific signalling (Cao et al., 2014; Miya et al., 2007; Sánchez‐Vallet et al., 2013). This signalling induces the activation of several plant defence mechanisms, including the accumulation of reactive oxygen species (ROS) and cell wall deposits, such as lignin and callose, that provide cell wall reinforcements (van den Burg et al., 2007; Doehlemann & Hemetsberger, 2013; Kaku et al., 2006; Mentlak et al., 2012).

To counter chitin‐triggered immunity, several effectors have been described in phytopathogenic fungi that play roles in avoiding chitin oligosaccharide recognition by plant receptors. One of these effectors is Avr4, a *Cladosporium fulvum* apoplastic effector, which protects fungal cell wall chitin from the action of plant chitinases released during the infection process (Bolton et al., 2008; van den Burg et al., 2007). Other effectors include Ecp6 and Slp1, proteins secreted by *C. fulvum* and *Magnaporthe oryzae*, respectively. These proteins sequester the free chitin oligosaccharides released as a consequence of the activity of plant chitinases, thus avoiding their recognition by the host. Another mechanism involved in the suppression of chitin‐triggered immunity is the action of the chitin deacetylase (CDA) enzyme. CDA is a widely conserved enzyme that catalyses the hydrolysis of the acetamido groups of *N*‐acetylglucosamine in chitin, promoting their conversion to chitosan, a glucosamine polymer and deacetylated chitin derivative that shows a considerably lower degree of immune elicitation than chitin (Mochizuki et al., 2011; Sánchez‐Vallet et al., 2013; Xi et al., 2014).

The suppression of PAMP‐triggered immunity by powdery mildew fungi has been a poorly investigated issue despite the fact that their nature as obligate biotrophs implies that the suppression of host defensive response activation should be a key aspect of their physiology. The first evidence in this regard has been the recent identification of effectors with chitinase activity (EWCAs), a family of conserved chitinases, secreted mainly by hyphae, that suppress chitin signalling by catalysing immunogenic chitooligosaccharides (Martínez‐Cruz et al., 2021). In the haustorial transcriptome of *P. xanthii*, a unigene encoding a small secreted protein without an annotated function but predicted by protein modelling to be a putative lytic polysaccharide monooxygenase (LPMO) with a chitin‐binding domain, was found among the top 50 expressed genes; it was the most highly expressed gene among those encoding proteins specifically expressed in the haustorium (Polonio, Seoane, et al., 2019). LPMOs are a class of recently characterized enzymes that are able to oxidize different recalcitrant polysaccharides, including chitin (Hemsworth et al., 2015; Vaaje‐Kolstad et al., 2010). Chitin LPMOs act on the crystalline chitin surface, introducing chain breaks and generating oxidized chain ends (Vaaje‐Kolstad et al., 2010). These enzymes are part of a pool of enzymes that different organisms secrete to obtain energy from dead biomass (Hemsworth et al., 2014). However, to date, LPMOs have not been reported in plant‐pathogenic or biotrophic fungi. Considering the putative role of this protein in chitin modification, as well as its high and exclusive expression in the haustorium, in this work we analysed the role of this putative LPMO in powdery mildew pathogenesis using computational approaches, experiments with purified recombinant proteins, and RNAi silencing experiments. Our results suggest that this effector could play a role in the catalysis of the chitin oligosaccharides released during the development of haustoria by plant endochitinases, thus avoiding the perception of chitin by the host plant and thereby allowing the development of haustoria inside plant epidermal cells.

## 2 RESULTS

### 2.1 The protein folding of PHEC27213 is similar to an LPMO and it contains a putative chitin‐binding domain

**FIGURE 1:**
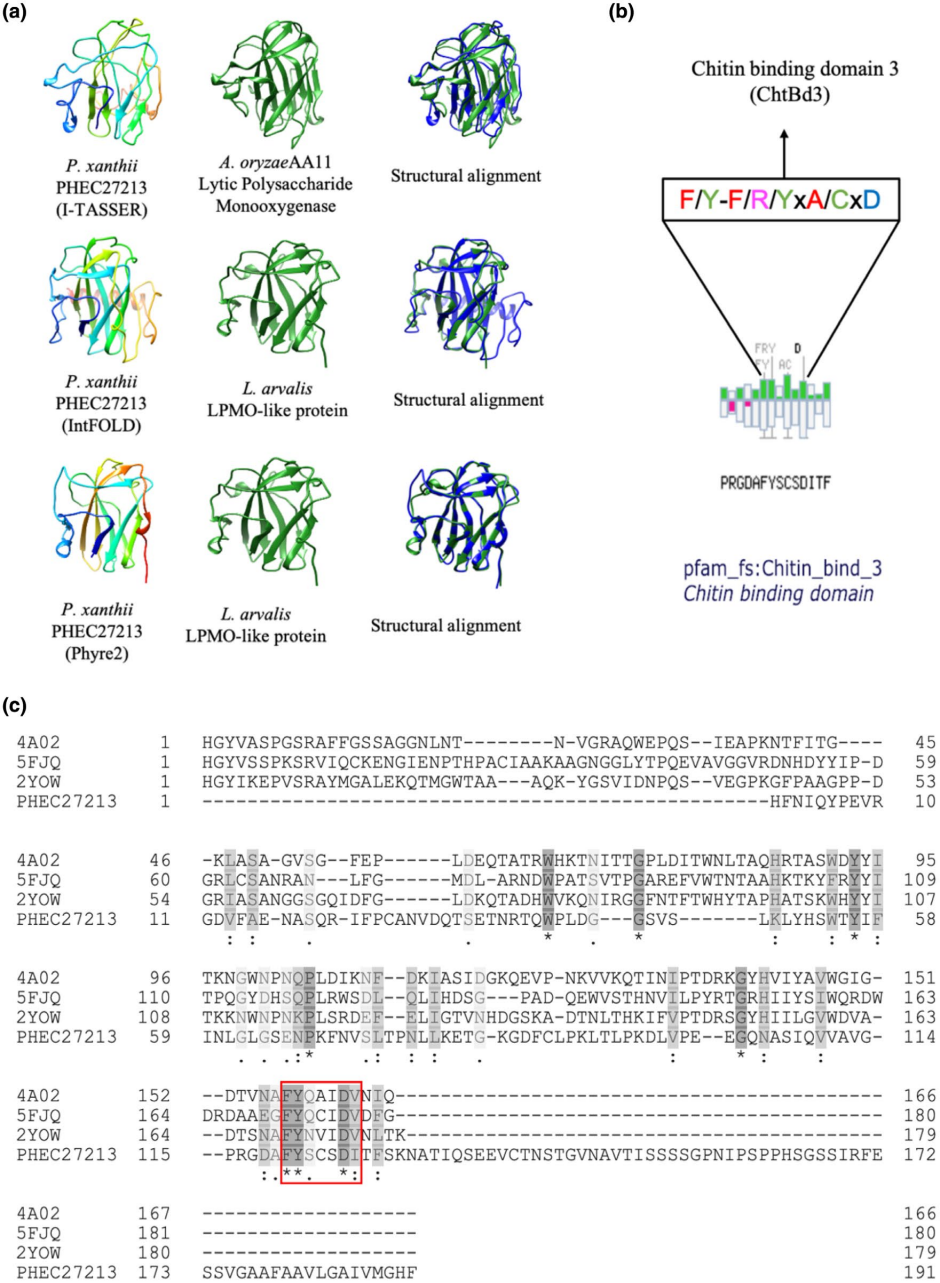
Main features predicted for the PHEC27213 protein. (a) Predicted three‐dimensional (3D) models of *Podosphaera xanthii* PHEC27213 performed by the I‐TASSER, IntFOLd, and Phyre2 servers, as well as the most similarly folded proteins. The structural alignments of both 3D models are also shown. (b) Amino acid sequence of the putative chitin‐binding domain of PHEC27213 predicted by MotifScan. Conserved residues are shown in the box. (c) Sequence alignment of PHEC27213 and several AA10 chitin lytic polysaccharide monooxygenase (LPMO) proteins retrieved from the Protein Data Bank, 5FJQ from *Cellvibrio japonicus*, 4A02 from *Enterococcus faecalis*, and 2YOW from *Bacillus amyloliquefaciens*. The amino acid similarity is shown in grey, with the darkest grey amino acids being the most similar. Conserved amino acids are marked with an asterisk. The putative chitin‐binding domain, corresponding to several conserved residues from CBM33 of the AA10 chitin LPMOs, is shown in a red box

The unigene PHEC27213 was selected from the *P. xanthii* haustorial transcriptome because it was the most highly expressed, haustorium‐specific gene encoding a secreted protein. However, because PHEC27213 lacks an annotated function or domains, the amino acid sequence corresponding to the mature protein, without the signal peptide, was used to perform 3D modelling using the I‐TASSER, Phyre2, and IntFOLD servers to elucidate the putative function of PHEC27213. In all cases, the resulting 3D models were similar (Figure 1a). These models were used as templates to perform protein fold recognition analyses. The I‐TASSER model matched with high confidence with an AA11 LPMO from *Aspergillus oryzae* (PDB code 4MAH) (Figure 1a, Table 1), whereas the Phyre2 and IntFOLD models matched with high confidence with an LPMO‐like protein from *Laetisaria arvalis* (PDB code 6IBH) (Figure 1a, Table 1). However, there was only 15.4% and 21.8% sequence identity between PHEC27213 and the *A. oryzae* and *L. arvalis* proteins, respectively (Figure S1).

**TABLE 1:** Main quality scores of the 3D protein models obtained for PHEC27213 using different servers

Server	Model quality score^a^	Structural analogues^b^	TM‐score^c^	Coverage^d^
I‐TASSER	−0.70	AA11 LPMO from *A. oryzae* (4MAH)	0.715	0.764
IntFOLD	0.463	LPMO‐like from *L. arvalis* (6IBH)	0.721	0.733
Phyre2	–	LPMO‐like from *L. arvalis* (6IBH)	97.5%	0.670

^a^ In I‐TASSER, this value is the C‐score. C‐score is in the range of −5 and 2, where a C‐score of higher value signifies a model with a high confidence. In IntFOLD, the global model quality scores range between 0 and 1, where scores less than 0.2 indicate there may be incorrectly modelled domains and scores greater than 0.4 generally indicate more complete and confident models. This value is not available in Phyre2.

^b^ Protein structurally closest to the corresponding PHEC27213 model according to protein fold recognition analysis. The corresponding PDB (Protein Data Bank) code is shown in parentheses.

^c^ TM‐score values are known standards for measuring the structural similarity between two structures, which are usually used to measure the accuracy of structure modelling when the native structure is known. In I‐Tasser and IntFOLD, TM‐scores are in the range of 0 to 1, being 1 a perfect match between models. In Phyre2, TM‐scores range between 0% and 100%.

^d^ Coverage represents the coverage of the alignment by TM‐align and is equal to the number of structurally aligned residues divided by length of the query protein.

On the contrary, it was not possible to detect any complete carbohydrate‐binding module (CBM) characteristic of the LPMO proteins using the Pfam or dbCAN2 servers. However, using MotifScan software, a putative chitin‐binding domain 3 (Pfam ID = Chitin_bind_3) was located from amino acids 115 to 128, corresponding to some residues of CBM33 from the AA10 LPMO (Figure 1b,c).

Thus, PHEC27213 appears to be a protein with a typical LPMO histidine brace and folding similar to LPMO proteins, while it lacks the full CBM domain that is present in canonical LPMOs; instead, it has only a few residues of this domain, which seem to be related to chitin binding. The presence of this putative chitin‐binding domain and the specific expression of this protein in the haustorium suggest that PHEC27213 could interact with the chitin from the haustorial cell wall of *P. xanthii*.

### 2.2 PHEC27213 shows chitin‐binding activity and breaks chitin into small oligosaccharides

**FIGURE 2:**
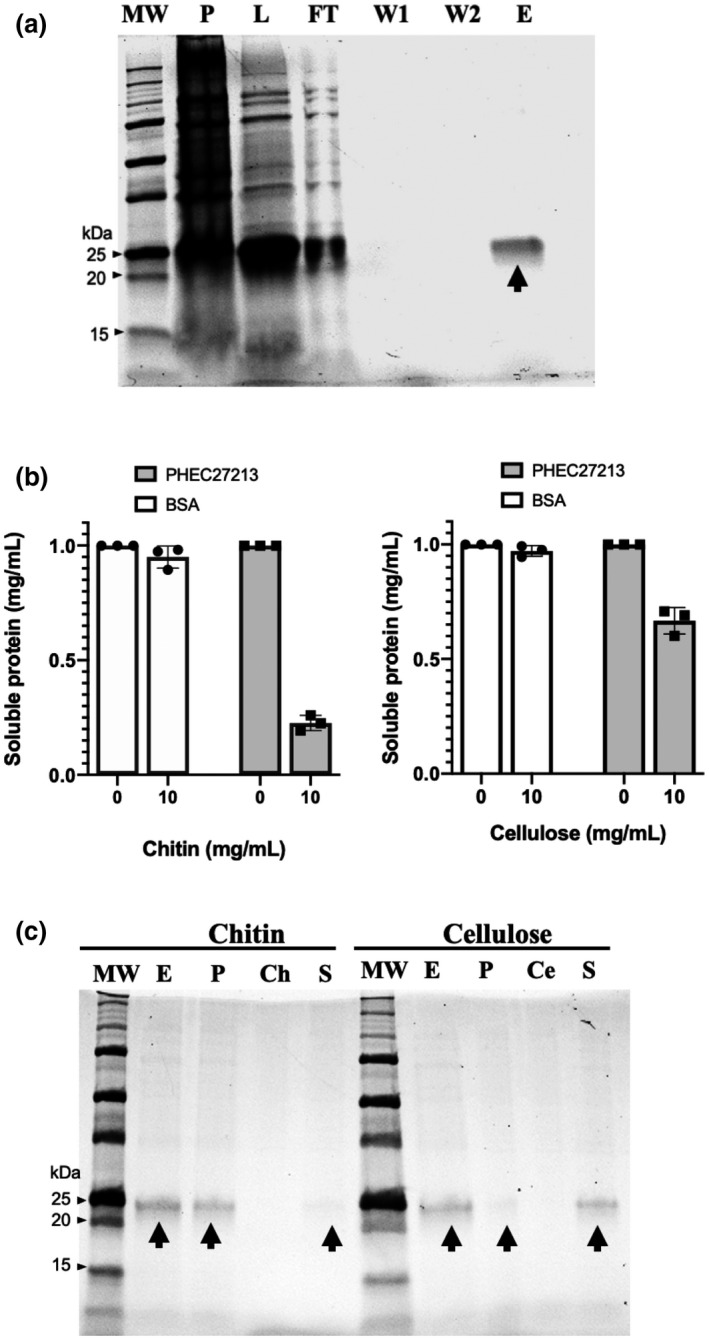
Polysaccharide‐binding activity of His‐tagged PHEC27213. (a) In vitro expression and purification of His‐tagged PHEC27213 protein. This protein was expressed in the *Escherichia coli* Bl21‐CodonPlus‐RIL and purified using Protino Ni‐TED 2000 Packed Columns (Macherey‐Nagel GmbH & Co). The image shows the different steps of protein purification visualized using Mini‐PROTEAN Stain‐Free Precast Gels (Bio‐Rad) in a ChemiDoc XRS + system (Bio‐Rad). The arrow indicates the band corresponding to the soluble purified protein. Lanes are MW (protein marker), Precision Plus Protein Unstained Standard (Bio‐Rad); P, pellet sample; L, supernatant sample after cell lysing; FT, discarded flow‐through sample after protein‐column binding; W1, first wash sample; W2, second wash sample; E, eluted soluble protein sample. (b) Polysaccharide‐binding activity of His‐tagged PHEC27213 protein. Quantification of soluble proteins (supernatant fraction) after incubation of soluble PHEC27213 (1 mg/ml) for 60 min with colloidal chitin or cellulose (10 mg/ml). Bovine serum albumen (BSA) was used as a negative control. Bars indicate the standard error of three technical replicates from three different experiments. (c) Western blot analysis of the His‐tagged PHEC27213 protein from the supernatant samples of the polysaccharide‐binding assay described in (b). The lanes are MW (protein marker), Precision Plus Protein Unstained Standard (Bio‐Rad); E (eluted protein), soluble PHEC27213 (1 mg/ml) taken from the final elution step after protein expression; P (pellet fraction), presence of PHEC27213 in the pellet after incubation with colloidal chitin or cellulose; Ch (chitin), colloidal chitin solution; Ce (cellulose), cellulose solution; S (supernatant fraction), presence of PHEC27213 in the supernatant after incubation with colloidal chitin or cellulose. Arrows indicate the bands corresponding to the PHEC27213 protein

To validate the function of PHEC27213, the protein was expressed in vitro as a 6 × His‐tagged fusion in the *Escherichia coli* Bl21‐CodonPlus‐RIL. PHEC27213 was predominantly found in the soluble fraction and was obtained after purification by immobilization on a nickel affinity column with a yield of 1.34 mg/ml of soluble protein (Figure 2a). The polysaccharide‐binding ability of the PHEC27213 protein was studied via a binding assay using colloidal chitin and cellulose as polysaccharides and bovine serum albumin (BSA) as a negative control. Only approximately 20% of the total soluble protein was present in the supernatant after exposure to chitin and approximately 70% was present after exposure to cellulose, indicating a high binding of PHEC27213 to chitin. By contrast, BSA was fully recovered from the supernatant, indicating the absence of binding (Figure 2b). These samples were also analysed by western blot analysis. In the presence of colloidal chitin, most of the PHEC27213 was retained in the pellet. However, while PHEC27213 was also observed in the pellet in the presence of cellulose, the protein was mainly detected in the supernatant (Figure 2c).

**FIGURE 3:**
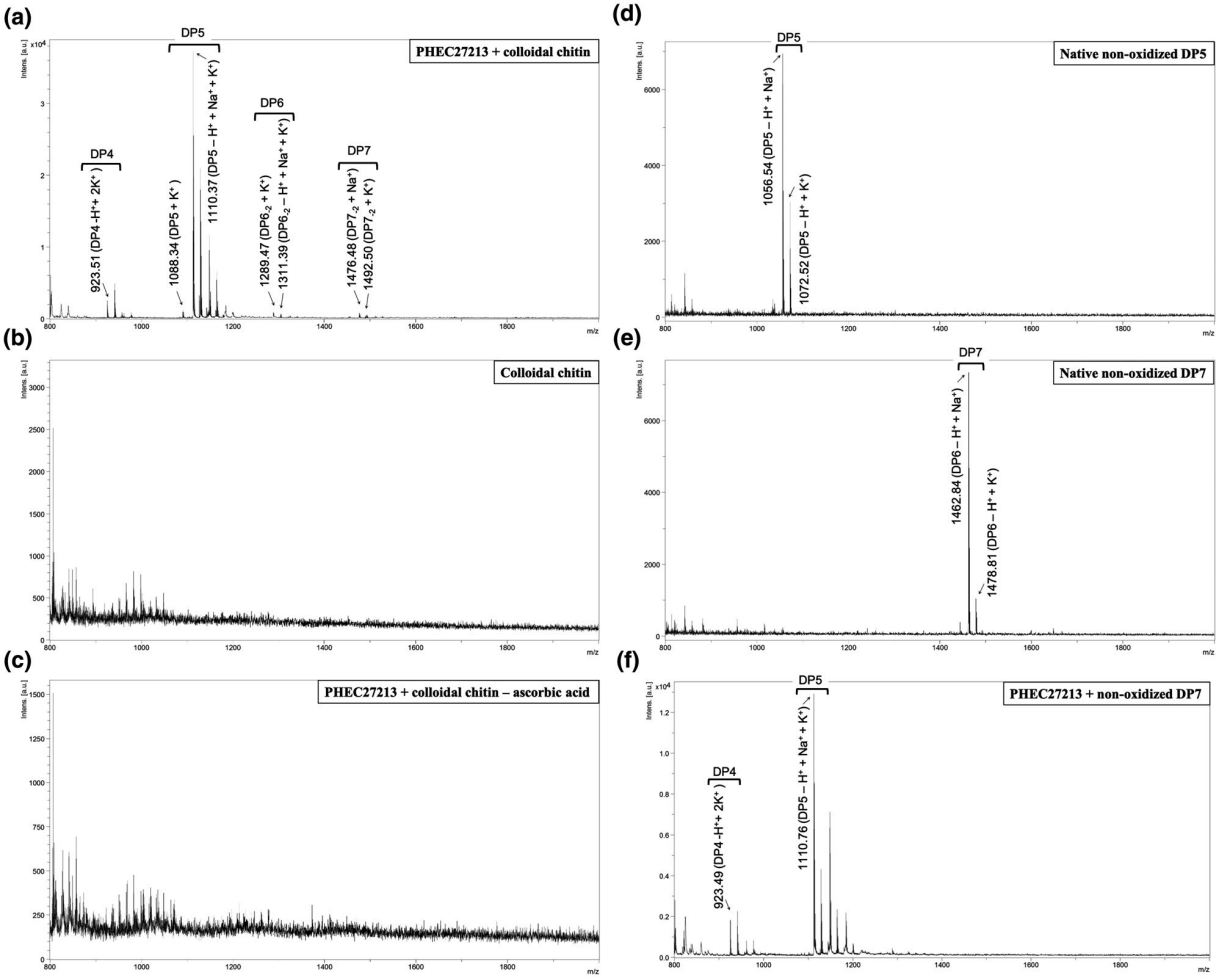
Lytic polysaccharide monooxygenase (LPMO) activity of His‐tagged PHEC27213 on different substrates. The protein PHEC27213 (1 μM) was incubated with the corresponding substrates in the presence of 1 mM ascorbic acid in 0.1 M sodium phosphate buffer (pH 7.0) at 37 ºC overnight. After incubation, the reaction products were analysed by MALDI‐TOF‐MS. The peaks corresponding to the adducts of oxidized chitin oligosaccharides were annotated according to Vaaje‐Kolstad et al. (2010) and Hemsworth et al. (2014). (a) MALDI‐TOF‐MS analysis of the products of an LPMO reaction with colloidal chitin (2 mg/ml) as the substrate. (b, c) Analysis of the products of the same reaction without PHEC27213 (b) or without ascorbic acid (c). (d‐e) MALDI‐TOF‐MS analyses of chitooligosaccharides. (d) Native nonoxidized DP5 (penta‐*N*‐acetylchitopentaose *m*/*z* = 1,033.98). (e) Native nonoxidized DP7 (hepta‐*N*‐acetylchitoheptaose *m*/*z* = 1,440.36). (f) Analysis of the products of an LPMO reaction with native nonoxidized DP7 as a substrate

The putative function of PHEC27213 was also studied in terms of chitin and cellulose degradation. For this purpose, the protein was incubated overnight with colloidal chitin or cellulose and ascorbic acid as a reducing agent, and after incubation the presence of free oligosaccharides was analysed by matrix‐assisted laser desorption ionization‐time of flight‐mass spectrometry (MALDI‐TOF‐MS). In the reaction of PHEC27213 with chitin, small oligosaccharides were detected (Figure 3a), whereas in the same reaction without PHEC27213 (Figure 3b) or without ascorbic acid (Figure 3c), it was not possible to detect small chitin oligosaccharides; because of their low solubility, longer oligosaccharides were never observed. In the reaction with chitin, among the small oligosaccharides, those with a degree of polymerization of 5 (DP5) were the most highly represented (Figure 3a). In addition, smaller peaks corresponding to DP4 and very small peaks corresponding to DP6 and DP7 were also detected. In the cases of DP6 and DP7, they presented a difference of −2 *m*/z, which could be due to the oxidation of the free C4 in the terminal monosaccharide (Figure S2). To check the number of oxidations that may occur during chitin catalysis by PHEC27213, the unoxidized standard oligosaccharide corresponding to the most predominant peak detected after enzymatic assays with PHEC27213, that is, DP5, was also analysed by MALDI‐TOF‐MS, which showed an *m*/*z* difference that corresponded with a single oxidation (Figure 3d). Moreover, to test the ability of PHEC27213 to catalyse oligosaccharides, a reaction with unoxidized DP7 (Figure 3e) was also carried out. An analysis of the reaction products showed that indeed PHEC27213 was able to release, predominantly, DP5 oligosaccharides (Figure 3f). In the case of cellulose, no peaks corresponding to oligosaccharides released from cellulose could be detected, indicating that PHEC27213 did not catalyse cellulose (Figure S3). These results revealed the ability of the PHEC27213 protein to bind and catalyse chitin via a single oxidation. With such activity and its predicted structure as an LPMO, the protein was renamed PxLPMO1 (*P. xanthii* lytic polysaccharide monooxygenase 1).

### 2.3 Molecular docking suggests a binding site for the chitin heptamer near the histidine brace active site

**FIGURE 4:**
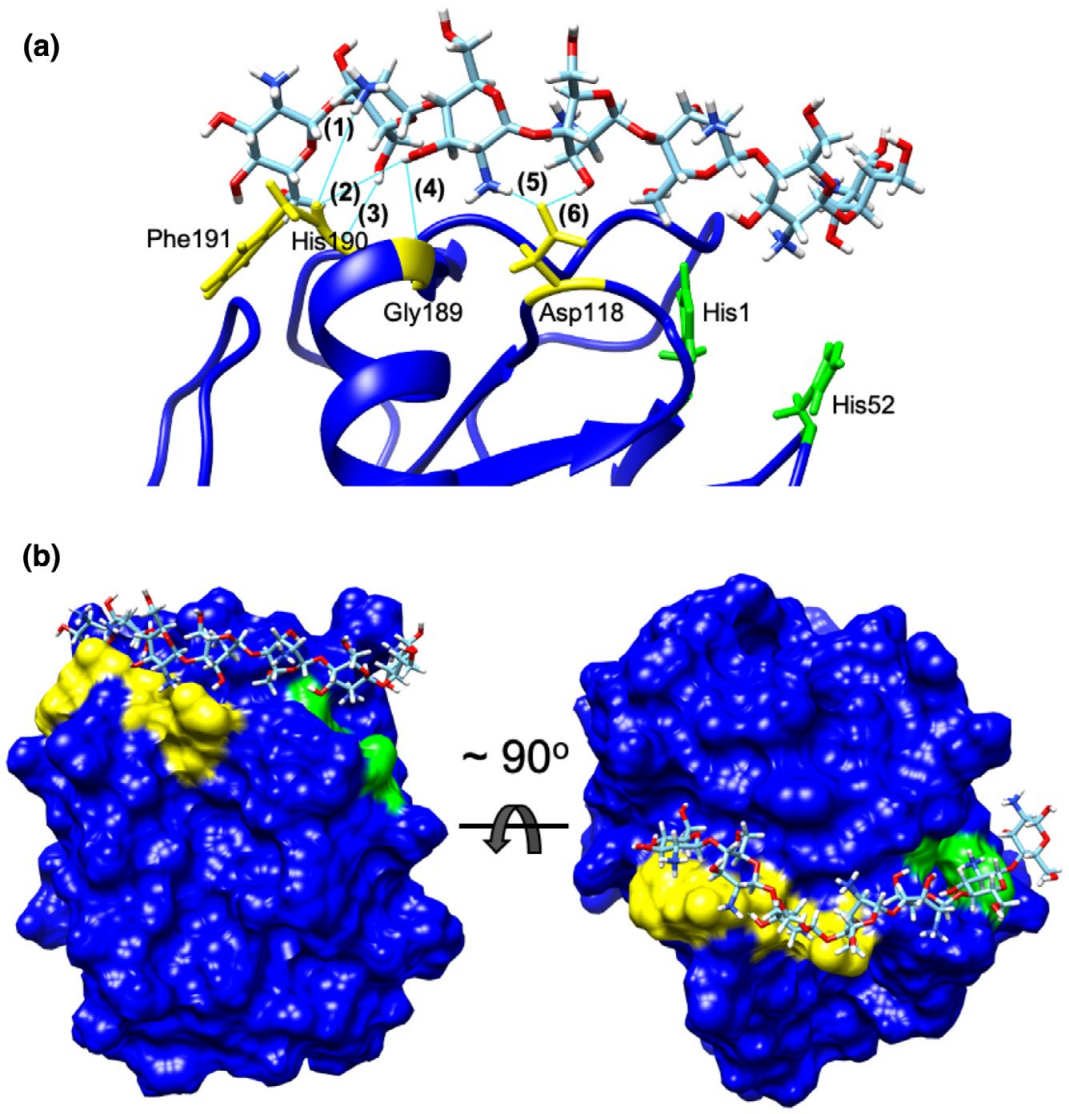
Molecular docking of chitin heptamer (DP7) to PxLPMO1. Docking analysis was performed with SwissDock software using the 3D model of PxLPMO1 predicted by IntFOLD and the DP7 molecule and visualized using UFSC Chimera software. (a) Docking results of putative PxLPMO1‐DP7 binding. DP7 is proposed to bind to PxLPMO1 via six hydrogen bonds (cyan lines) that involve (yellow) Phe191 (1:1.93 Å; 2:1.86 Å), His190 (3:2.05 Å), Gly189 (4:2.79 Å), and Asp118 (5:1.77 Å; 6:2.11 Å). (b) Panoramic view of the PxLPMO1 protein with the DP7 molecule docked in the proposed binding site. DP7 binds to PxLPMO1 via residues 191, 190, 189, and 118 (yellow), which allow the interaction of the chitin heptamer with residues 1 and 52 of the histidine brace active site (green)

The putative chitin‐binding site was computationally assessed by molecular docking using the best 3D model of PHEC27213 (PxLPMO1), that is, that predicted by IntFOLD (Table 1), and the 3D model of the chitin heptamer (DP7). Molecular docking analysis using the SwissDock server resulted in the identification of 31 clusters at different sites of the protein. Among them, cluster 24 showed the best “Full Fitness” parameter (−544.95) and the lowest ΔG (−12.97 kcal/mol). This putative binding site is formed by Asp118, Gly189, His190, and Phe191, which can form six putative hydrogen bonds with DP7, two with the amino group and the hydroxyl group of Phe191 ([1] 1.93 Å, [2] 1.86 Å), one with the hydroxyl group of His190 ([3] 2.05 Å), one with the hydroxyl group of Gly189 ([4] 2.79 Å), and two with the amino group and the hydroxyl group of Asp118 ([5] 1.77 Å and [6] 2.11 Å) (Figure 4a). Three of these residues (Gly189, His190, and Phe191) are located in the C‐terminal domain of the protein, while Asp118 is located near the putative chitin‐binding motif predicted by MotifScan (residues 120–126). The binding site identified by molecular docking is located very close to the histidine brace (His1 and His52), the active site of LPMO enzymes (Figure 4b), with the closest oxidation site being the C1 of DP5 (Figure 4a), which is consistent with the predominant release of DP5 observed in the enzymatic assays.

### 2.4 *PxLPMO1* is coexpressed with two plant endochitinase genes

**FIGURE 5:**
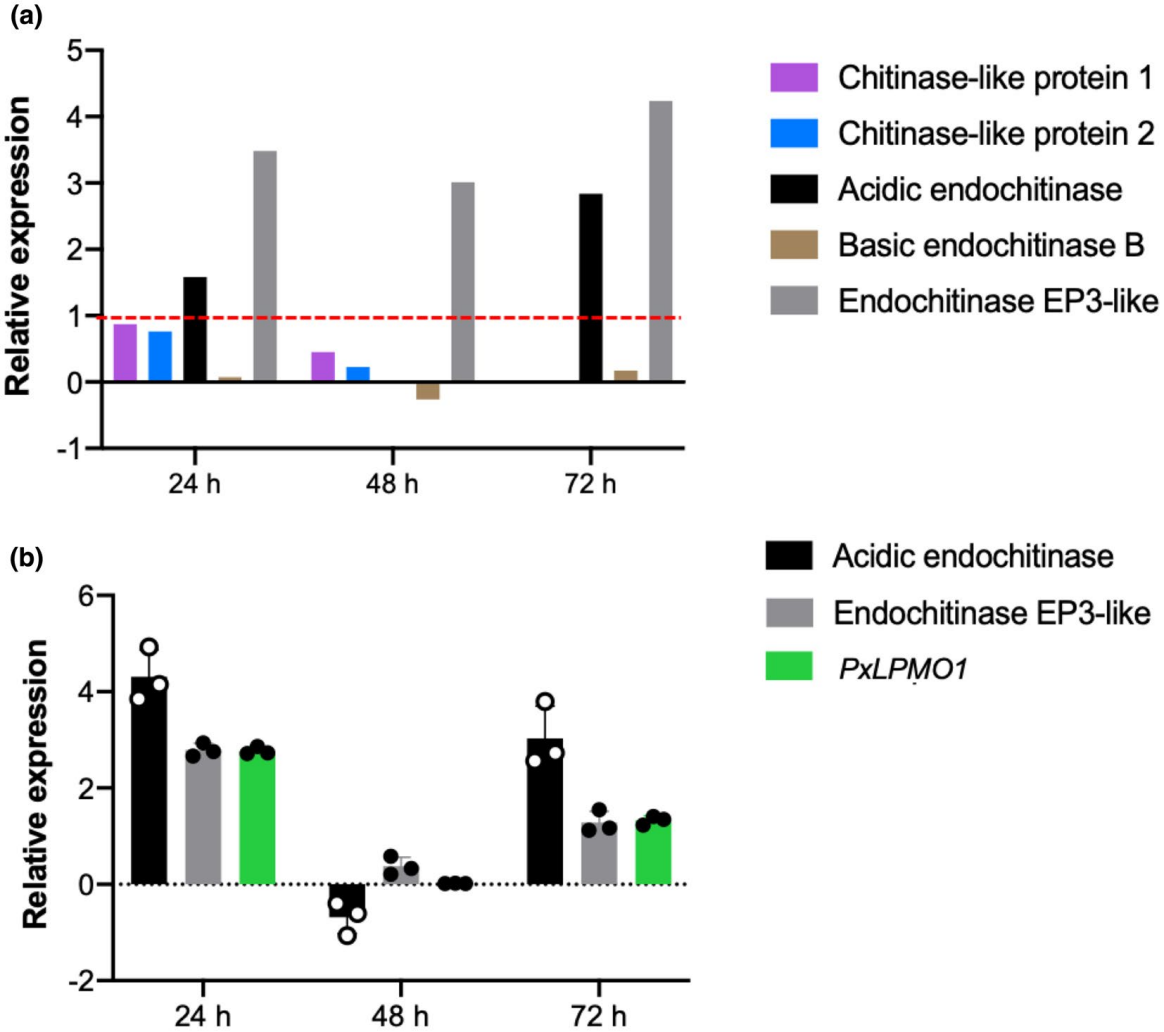
Expression pattern analysis of *Cucumis melo* chitinases and *Podosphaera xanthii PxLPMO1* during the first stages of infection. (a) Expression of melon chitinases in response to *P. xanthii* infection extracted from previous RNA‐Seq data (Polonio, Pineda, et al., 2019). The discontinuous red line indicates the considered significance threshold for induced expression (log_2_(fold change) > 1). (b) Relative expression of *PxLPMO1* and highly expressed host endochitinases analysed by quantitative reverse transcription PCR. Total RNA was isolated from melon cotyledons at different time points after inoculation with *P. xanthii*. The expression of plant endochitinases is represented as the log_2_(FC) relative expression (infected plants/control plants), whereas the expression of the *PxLPMO1* gene was normalized to the transcription of the endogenous control, the elongation factor 1 gene *PxEF1* (MK249653). Bars show the standard error of three technical replicates from three different experiments

To elucidate the physiological function of *PxLPMO1*, its expression was studied during the first 72 hr of infection. In addition, the expression of host chitinase genes was also analysed during the same period of interaction. For this purpose, data were first extracted from an RNA‐Seq analysis performed previously (Polonio, Pineda, et al., 2019). Five different chitinases were identified: chitinase‐like protein 1 (KAA0044632.1), chitinase‐like protein 2 (XP_008438940.1), acidic endochitinase (XM_008439336.2), basic endochitinase B (XP_008448502.1), and acidic EP3‐like endochitinase (XM_008446389.2). Among them, only acidic endochitinase (XM_008439336.2) and acidic EP3‐like endochitinase (XM_008446389.2) were strongly expressed in *P. xanthii*‐infected melon leaves (Figure 5a). To validate the RNA‐Seq data, the expression patterns of these melon endochitinases were analysed by quantitative reverse transcription PCR (RT‐qPCR). In these experiments, the expression of *PxLPMO1* was also studied. The results showed that host and pathogen genes presented similar expression patterns during the course of infection (Figure 5b). Moreover, the Pearson's correlation coefficients obtained for the expression of acidic endochitinase and acidic EP3‐like endochitinase in relation to the expression of *PxLPMO1* were 0.914 and 0.991, respectively, demonstrating that host endochitinases and *PxLPMO1* are coexpressed during the first stages of *P. xanthii* infection in melon plants.

### 2.5 RNAi silencing of the *PxLPMO1* gene reduces *P. xanthii* development and activates an oxidative burst

**FIGURE 6:**
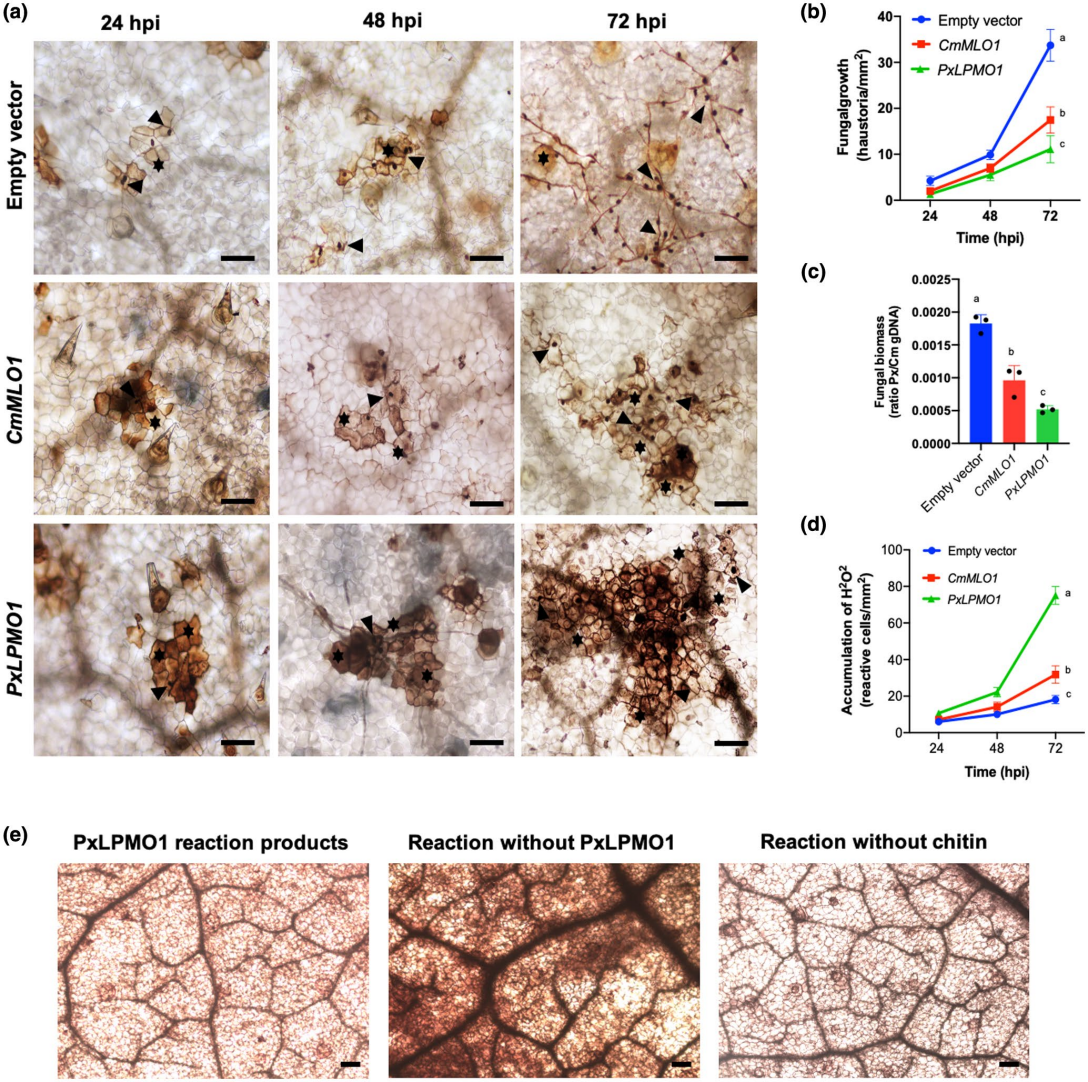
Effect of silencing *PxLPMO1* on fungal growth and oxidative burst. RNA interference (RNAi) silencing was carried out by *Agrobacterium tumefaciens*‐mediated host‐induced gene silencing. An empty vector was used as a negative control, and RNAi silencing of the melon *CmMLO1* gene was used as a positive control for RNAi‐induced resistance. (a) Visualization of fungal structures and oxidative bursts. Detection of hydrogen peroxide was performed by the 3,3′‐diaminobenzidine (DAB) uptake method. Pictures were taken at 24, 48, and 72 hr after inoculation with *Podosphaera xanthii*. Arrowheads indicate penetration points corresponding to haustoria, and asterisks indicate reactive epidermal cells with H_2_O_2_ accumulation. Bars = 100 μm. (b) Estimation of fungal growth by haustorial counting. The growth of *P. xanthii* is expressed as the number of haustoria per mm^2^ of transformed tissue. The values are the means of 30 samples from three independent experiments ± standard error. (c) Molecular estimation of *P. xanthii* growth by quantitative PCR. Agroinfiltrated melon cotyledons taken 72 hr after pathogen inoculation were used for isolation of genomic DNA. The genomic DNA ratio of *P. xanthii* to melon cotyledons (Px/Cm gDNA) was used as an indicator of fungal biomass after amplification of the P. xanthii *β‐tubulin* and *Cucumis melo* actin‐7 genes (Table S2). Bars indicate the means ± standard error of three technical replicates from three different DNA samples each derived from five pooled cotyledons. (d) Time course analysis of the accumulation of H_2_O_2_ in melon cotyledons. Detection of H_2_O_2_ was performed by the DAB uptake method. The reactive epidermal cells were identified as those containing brown‐red precipitates (Figure 5a, asterisks). Data represent the number of reactive cells per mm^2^ and are the means of 30 samples from three independent experiments ± standard error. Values with different letters in (b), (c), and (d) are significantly different at *p* = .05 according to Fisher's least significant difference test (LSD). (e) Effect of the infiltration of the products from the PxLPMO1 (PHEC27213) enzymatic reaction on the oxidative burst. The supernatants from the reaction products described in Figure 3a were infiltrated into melon cotyledons, and then the samples were processed for detection of H_2_O_2_ by the DAB uptake method. As positive and negative controls for the production of oxidative burst in leaf tissue, the products of the same reactions without PxLPMO1 or without colloidal chitin were used, respectively

The high expression of *PxLPMO1*, which was the 13th most highly expressed gene in the haustorium and the most highly expressed gene among the genes encoding haustorium‐specific secreted proteins (Polonio, Seoane, et al., 2019), suggested that it plays an important role in *P. xanthii* biology. To validate the role of *PxLPMO1*, the *Agrobacterium tumefaciens*‐mediated host‐induced gene silencing (ATM‐HIGS) assay was used. To quantify fungal growth after gene silencing, two approaches were used: haustorial counts by light microscopy and a molecular approach by quantitative PCR (qPCR). The activation of plant defence responses was also studied. In this regard, the production of reactive oxygen species, such as hydrogen peroxide (H_2_O_2_), was histochemically examined. The efficacy of ATM‐HIGS was studied by RT‐qPCR, showing that the levels of the *CmMLO1* (the positive control for RNAi‐induced resistance) and *PxLPMO1* transcripts decreased by approximately 50% during RNAi gene silencing experiments (Figure S4). As shown in Figure 6, after silencing *PxLPMO1*, the development of *P. xanthii* was clearly altered and delayed, and this decrease was evident 72 hr after inoculation compared with the negative control (empty vector). In parallel, a strong accumulation of H_2_O_2_ was observed that was even higher than that observed in the samples corresponding to the *CmMLO1* RNAi‐positive control (Figure 6a). As observed in the figure, the quantification of fungal growth by haustorial counts (Figure 6b) and the estimation of fungal biomass by qPCR (Figure 6c) showed a dramatic reduction in fungal development in *PxLPMO1*‐silenced cotyledons compared to that in the negative control (empty vector), with both assays achieving similar percentages of growth reduction of approximately 70% at 72 hr after pathogen inoculation. Regarding the activation of the oxidative burst, silencing of *PxLPMO1* resulted in a strong accumulation of H_2_O_2_ (Figure 6d), suggesting the likely activation of chitin‐triggered immunity in *PxLPMO1*‐silenced tissues. Accordingly, the infiltration of leaf tissue with the supernatant of the PxLPMO1 reaction products described above showed no activation of the oxidative burst. By contrast, the oxidative burst was rapidly and strongly activated by the infiltration of the supernatant of the reaction mixture without PxLPMO1 (Figure 6e).

### 2.6 RNAi silencing of the *PxLPMO1* gene activates chitin‐triggered immunity

**FIGURE 7:**
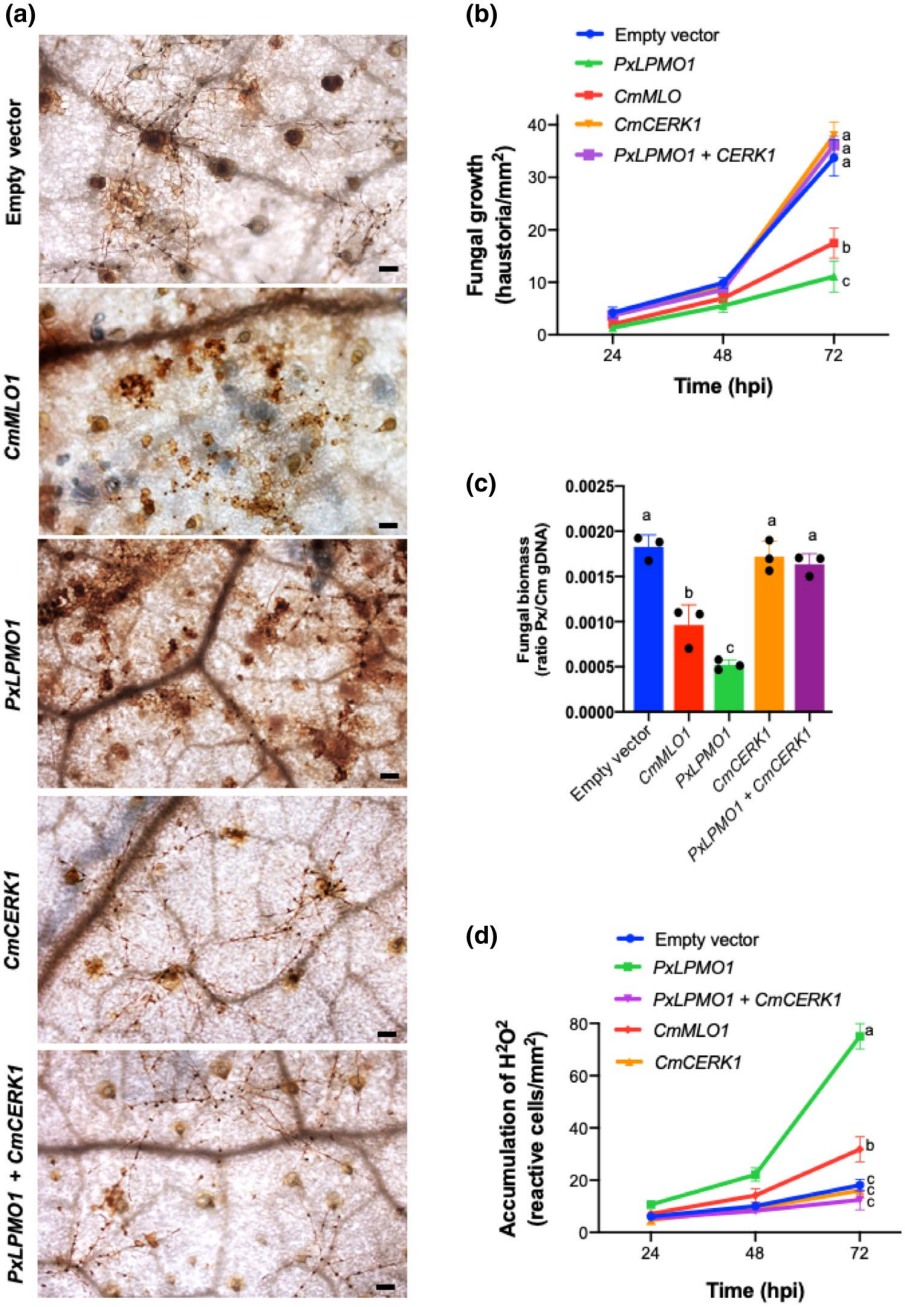
Effect of co‐silencing of *PxLPMO1* and the melon chitin receptor kinase gene *CmCERK1* on fungal growth and the oxidative burst. RNAi silencing was carried out by *Agrobacterium tumefaciens*‐mediated host‐induced gene silencing. The empty vector was used as a negative control, and RNAi silencing of the melon *CmMLO1* gene was used as a positive control. (a) Visualization of fungal structures and oxidative bursts. Detection of H_2_O_2_ was performed by the 3,3′‐diaminobenzidine uptake method. Pictures were taken at 24, 48, and 72 hr after inoculation (hpi) with *Podosphaera xanthii*. Fungal hyphae and haustoria (black dots) are easily recognized. The brown colour indicates the presence of reactive epidermal cells with H_2_O_2_ production. Bars = 100 μm. (b) Estimation of fungal growth by haustorial counting. The growth of *P. xanthii* is expressed as the number of haustoria per mm^2^ of transformed tissue. The values are the means of 30 samples from three independent experiments ± standard error. (c) Molecular estimation of *P. xanthii* growth. Cotyledon samples were taken at 72 hpi and were used for the isolation of genomic DNA. The ratios of *P. xanthii* to melon cotyledon genomic DNA (Px/Cm gDNA) were determined by quantitative PCR as indicated in Figure 5c. Bars indicate the means ± standard errors of three technical replicates from three different DNA samples each derived from five pooled cotyledons. (d) Time course analysis of H_2_O_2_ production by epidermal cells. Detection of H_2_O_2_ was performed by the DAB uptake method. The reactive epidermal cells were identified as containing brown‐red precipitates (Figure 5a, asterisks). Data represent the number of reactive cells per mm^2^ and are the means of 30 samples from three independent experiments ± standard error. Different letters in (b), (c), and (d) indicate values significantly different at *p* = .05 according to Fisher's least significant difference test (LSD)

The ability of the purified PxLPMO1 protein to catalyse chitin combined with the fact that the supernatant of PxLPMO1 reaction products was not capable of triggering an oxidative burst in leaf tissue suggested that the activity of PxLPMO1 was related to preventing the activation of chitin‐triggered immunity. To conclusively determine this role of PxLPMO1, new RNAi silencing experiments, including the RNAi silencing of the melon chitin receptor kinase gene *CmCERK1*, were performed. The efficacy of ATM‐HIGS during co‐silencing was studied by RT‐qPCR, and similar to the single RNAi silencing experiments the transcript levels of *CmMLO1*, *PxLPMO1*, and *CmCERK1* decreased by approximately 50%–60% (Figure S4). Regarding the phenotypes, as expected, co‐silencing of the *PxLPMO1* and *CmCERK1* genes restored the original phenotype, that is, there was normal development of *P. xanthii* and a low level of reactive epidermal cells compared to that found with only *PxLPMO1* silencing, similar to the negative control (empty vector; Figure 7a). The quantification of fungal growth by both haustorial counts (Figure 7b) and qPCR (Figure 7c) supported the phenotypes observed by light microscopy; *P. xanthii* development after co‐silencing of the *PxLPMO1* and *CmCERK1* genes was virtually indistinguishable from that of the negative control and significantly different from that obtained with the silencing of only *PxLPMO1*. Similarly, quantification of hydrogen peroxide production by epidermal cells showed that there was a basal level of reactive cells in co‐silenced tissues that was identical to the negative control (Figure 7d), which indicated that the oxidative burst typical of chitin‐triggered immunity was not activated in such circumstances.

### 2.7 *PxLPMO1* orthologues and putative LPMOs are present in many pathogenic ascomycete fungi and in other haustorium‐forming fungal pathogens

The presence of *PxLPMO1* orthologues was studied by BLASTp (BLAST + v. 2.7.1; *E* value < 1E−5) analysis using the deduced amino acid sequence of PxLPMO1 as a query sequence. As shown in Table 2, orthologous genes are widely present in the genomes of many pathogenic ascomycete fungi, mostly in animal and human pathogens, but also in plant and insect pathogens. Furthermore, this gene is also present in endophytic/endomycorrhizal fungi. The presence of *PxLPMO1* orthologues in saprophytic fungi is very limited in comparison with their wide presence in pathogenic and endophytic/endomycorrhizal fungi. However, it was surprising that no orthologues were found in other haustorium‐forming fungal pathogens.

**TABLE 2:** Fungal species assessed for the presence of *PxLPMO1* orthologues

Species	Pathogenicity	Identity (%)	Accession no.
Leotiomycetes			
*Rhynchosporium commune*	Plant pathogen	60	CZT02273.1
*Rhynchosporium secalis*	Plant pathogen	60	CZT53033.1
*Rhynchosporium agropyri*	Plant pathogen	61	CZT08592.1
*Cadophora* sp.	Plant pathogen	61	PVH76518.1
*Phialophora americana*	Animal/human pathogen	52	KIW70575.1
*Phialophora hyalina*	Endophytic/endomycorrhizal	55	RDL36398.1
*Hyaloscypha finlandica*	Endophytic/endomycorrhizal	52	ACP19527.1
*Hyaloscypha variabilis*	Endophytic/endomycorrhizal	59	PMD37570.1
*Coleophoma crateriformis*	Endophytic/endomycorrhizal	54	RDW78501.1
*Coleophoma cylindrospora*	Plant pathogen	60	RDW84639.1
*Duddingtonia flagrans*	Entomopathogen	53	RVD84252.1
*Dactylellina haptotyla*	Entomopathogen	48	EPS41873.1
*Glarea lozoyensis*	Saprophytic	54	XP_008087835.1
*Pezoloma ericae*	Endophytic/endomycorrhizal	53	PMD21535.1
*Meliniomyces bicolor*	Endophytic/endomycorrhizal	52	XP_024729819.1
Dothideomycetes			
*Cladophialophora yegresii*	Animal/human pathogen	52	XP_007754892.1
*Cladophialophora carrionii*	Animal/human pathogen	53	OCT50285.1
*Cladophialophora bantiana*	Animal/human pathogen	54	XP_016616728.1
*Cladophialophora psammophila*	Animal/human pathogen	54	XP_007747041.1
*Cladophialophora immunda*	Animal/human pathogen	46	XP_016253641.1
*Hortaea werneckii*	Animal/human pathogen	49	OTA23506.1
Eurotiomycetes			
*Chaetothyriales* sp.	Animal/human pathogen	50	RMZ91855.1
*Rhinocladiella mackenziei*	Animal/human pathogen	54	XP_013274494.1
*Arthrobotrys oligospora*	Entomopathogen	52	XP_011117840.1
*Exophiala aquamarina*	Animal/human pathogen	54	XP_013256137.1
*Exophiala dermatitidis*	Animal/human pathogen	50	XP_009158214.1
*Exophiala xenobiotica*	Animal/human pathogen	53	XP_013315436.1
*Fonsecaea erecta*	Animal/human pathogen	53	XP_013315436.1
*Fonsecaea multimorphosa*	Animal/human pathogen	54	XP_016636211.1
*Fonsecaea pedrosoi*	Animal/human pathogen	52	XP_013285390.1
Sordariomycetes			
*Ophiostoma piceae*	Plant pathogen	47	EPE09813.1
*Sporothrix insectorum*	Entomopathogen	52	OAA55257.1

With the absence of *PxLPMO1* orthologues in haustorium‐forming fungal pathogens after the first analysis, a more exhaustive search was performed using BLASTp (BLAST+ v. 2.7.1; *E* value < 1E−5) against the available proteomes of the powdery mildew fungi *Blumeria graminis* f. sp. *hordei* DH14, *Erysiphe necator*, *Erysiphe pulchra*, and *Golovinomyces cichoracearum*, as well as the proteomes of the rust fungi *Melampsora larici‐populina*, *Puccinia graminis* f. sp. *tritici*, *Puccinia triticina*, *Puccinia striiformis* f. sp. *tritici*, and *Puccinia sorghi*. Similarly, tBLASTn and BLASTn searches (BLAST+ v. 2.7.1; *E* value < 1E−5) were used to scan the newly assembled *P. xanthii* genomes for paralogs and other LPMO‐like proteins.

In all powdery mildew fungi examined, including *P. xanthii*, as well as in the poplar rust fungus *M. larici‐populi*, at least one PxLPMO1‐like protein was found (Figure S5 and Table 3). However, in the case of species of the genus *Puccinia*, none was found. These proteins showed common features with PxLPMO1 (similar folding, small size, presence of a signal peptide, the typical histidine brace, and the presence of conserved residues corresponding to chitin‐binding domain 3), but they only had sequence identities with PxLPMO1 of 20%–36% (Figure S5b and Table 3). This lack of identity is largely due to the high sequence variability present in the C‐terminal domain of PxLPMO1 and PxLPMO1‐like proteins (Figure S5c). Moreover, according to DeepLoc software predictions, only three of the proteins (*B. graminis* LPMO‐like 2, *E. pulchra* LPMO‐like 2, and *M. larici‐populina* LPMO‐like 1) were predicted to be secreted proteins and, according to PredGPI predictions, four of the proteins were predicted as highly probable (*E. pulchra* LPMO‐like 1) or probable (*P. xanthii* LPMO‐like 2 and *M. larici‐populina* LPMO‐like 2 protein) and two of them were predicted as weakly probable (*B. graminis* LPMO‐like 2 and *G. cichoracearum* LPMO‐like 2) to be GPI‐anchored proteins (Table 3). Interestingly, phylogenetic analysis of these proteins showed that the LPMO‐like proteins from haustorium‐forming fungal pathogens form an independent clade and are phylogenetically separated from bacterial AA10 and fungal AA11 chitin LPMOs (Figure S5d).

**TABLE 3:** Main features of LPMO‐like proteins present in *P. xanthii* and other haustorium‐forming fungal pathogens

Protein	Length (amino acids)^a^	Annotation	Accession number	Identity (%)^b^	Signal peptide^c^	Location^d^	GPI‐anchored^e^
*Podosphaera xanthii* LPMO1	191	Putative uncharacterized protein	MT234390	100	Yes	Extracellular	No
*Podosphaera xanthii* LPMO‐like 2	205	Uncharacterized protein	—	26.89	Yes	Membrane	Probable
*Erysiphe necator* LPMO‐like 1	195	Putative GPI‐anchored protein	KHJ34437	26.87	Yes	Cell membrane	No
*Blumeria graminis* LPMO‐like 1	201	GPI‐anchored protein	CCU83093	25.71	Yes	Cell membrane	No
*Blumeria graminis* LPMO‐like 2	201	Hypothetical protein	CCU79606	26.29	Yes	Extracellular	Weakly probable
*Golovinomyces cichoracearum* LPMO‐like 1	191	Uncharacterized protein	RKF73882	30.00	Yes	Cell membrane	No
*Golovinomyces cichoracearum* LPMO‐like 2	209	Uncharacterized protein	RKF80963	27.40	Yes	Cell membrane	Weakly probable
*Erysiphe pulchra* LPMO‐like 1	196	Uncharacterized protein	POS84877	36.65	Yes	Cell membrane	Highly probable
*Erysiphe pulchra* LPMO‐like 2	146	Hypothetical protein (partial)	POS88026	32.32	Yes	Extracellular	No
*Melampsora larici‐populina* LPMO‐like 1	151	Hypothetical protein	XP_007414865	18.63	Yes	Extracellular	No
*Melampsora larici‐populina* LPMO‐like 2	197	Hypothetical protein	XP_007411790	18.63	Yes	Cell membrane	Probable

^a^ Length of the mature protein (without signal peptide).

^b^ Identity of the haustorium‐forming fungal pathogen protein against *Podosphaera xanthii* LPMO1 protein (PxLPMO1).

^c^ Presence of signal peptide predicted by SignalP 4.1 server.

^d^ Prediction of final location of mature protein using DeepLoc server.

^e^ Prediction of GPI‐anchored proteins by PredGPI software.

Finally, BLASTn searches of the *P. xanthii* genomes showed the presence of two copies of *PxLPMO1* that were identical in the two available genomes. The paralog A (*PxLPMO1A*) that was the object of this study was found in the JACSEY010001314.1 and JAAAXZ010000022.1 scaffolds, while the paralog B (*PxLPMO1B*) was found in the JACSEY010001350.1 and JAAAXZ010000055.1 scaffolds. Compared to *PxLPMO1A*, *PxLPMO1B* showed seven nucleotide changes that caused four amino acid substitutions, including one in the signal peptide (F2S) and two in the highly variable C‐terminus (R188G and G194W) (Figure S5e).

## 3 DISCUSSION

As an obligate biotroph, *P. xanthii* requires living cells to complete its asexual life cycle, which ultimately implies that it must suppress the activation of plant defence responses. The mechanisms by which *P. xanthii* avoids recognition by the host remain largely unknown. For this purpose, *P. xanthii*, among its other abilities, has to hide its PAMPs or manipulate their detection to suppress the activation of PTI. These activities are most likely carried out by the secretion of effectors. In other fungal pathogens, such as *C. fulvum* or *M. oryzae*, effector proteins with the ability to suppress chitin‐triggered immunity have been described (Bolton et al., 2008; van den Burg et al., 2007; Jonge et al., 2010; Sánchez‐Vallet et al., 2013; Xi et al., 2014). In the case of *P. xanthii*, the recent discovery of an effector family with chitinase activity (EWCAs) has revealed a new mechanism to suppress chitin signalling that consists of the degradation of immunogenic oligosaccharides, effectors that are also present in other powdery mildews and many fungal pathogens (Martínez‐Cruz et al., 2021). However, in the case of haustorium‐forming pathogens, to date no specific mechanisms associated with the haustorium have been identified to suppress chitin‐triggered immunity, despite the fact that this ability should be essential for the survival of this cell because it is the fungal structure that maintains the most intimate relationship with the host cells (Bindschedler et al., 2009; Both et al., 2005; Martínez‐Cruz et al., 2014; Micali et al., 2011). In this regard, the absence of any known mechanism related to this purpose in the most extensively studied powdery mildew species, *Blumeria graminis*, is striking, even though several studies have indicated that numerous candidate effectors expressed in haustoria become engaged in a coevolutionary arms race with the innate immune system of the host (Hacquard, 2014; Pedersen et al., 2012). Thus, proteomic studies in *B. graminis* revealed how its haustoria are enriched with small secreted proteins, including proteins involved in carbohydrate metabolism (Bindschedler et al., 2009, 2011; Godfrey et al., 2009) that can act in a similar way.

The fact that chitin is a major component of the haustorial cell wall (Micali et al., 2011) suggests the importance of neutralizing the recognition of this polymer by the host for successful infection by haustorium‐forming fungal pathogens. The haustorial transcriptome of *P. xanthii* (Polonio, Seoane, et al., 2019) allowed us to identify an effector candidate, PHEC27213, that had typical effector features, such as no annotated function, a small size, and a wave‐like expression pattern (Hacquard, 2014; Pedersen et al., 2012). Furthermore, PHEC27213 was the most highly expressed, haustorium‐specific, putative secreted protein (Polonio, Seoane, et al., 2019), pointing to its key role in haustorial physiology. In this work, we performed a molecular characterization of PHEC27213 to elucidate its putative function as a chitin manipulation‐related protein.

The computationally predicted function of PHEC27213 showed a similar protein folding to an AA11 LPMO from *A. oryzae* and to an LPMO‐like protein from *L. arvalis*, indicating that it has an LPMO‐like structure. To date, LPMOs have been classified into seven families (AA9, AA10, AA11, AA13, AA14, AA15, AA16) according to their auxiliary activity (AA) in the CAZy database (Levasseur et al., 2013; Lombard et al., 2014). The fungal AA9 family members cleave cellulose and hemicellulose (Agger et al., 2014; Bennati‐Granier et al., 2015; Frommhagen et al., 2015; Phillips et al., 2011; Quinlan et al., 2011; Vu, Beeson, Phillips, et al., 2014). AA10 LPMOs have been described in bacteria, viruses, and eukaryotic organisms, cleaving both cellulose and chitin (Forsberg et al., 2014; Vaaje‐Kolstad et al., 2010). AA11 and AA13 are present exclusively in fungi and target chitin and starch, respectively (Hemsworth et al., 2014; Lo Leggio et al., 2015; Vu, Beeson, Span, et al., 2014). AA14, AA15, and AA16 have been recently described; fungal AA14 members show activity on cellulose fibres coated with xylan (Couturier et al., 2018), whereas AA15 is an LPMO active against cellulose and chitin and the first LPMO of animal (invertebrate) origin (Sabbadin et al., 2018). Finally, the last family discovered, AA16, is present in fungi and is active against cellulose (Filiatrault‐Chastel et al., 2019).

Although the presence of common features of LPMO enzymes, such as a similar protein folding and the typical histidine brace (Hemosworth et al., 2014), supported the putative function of PHEC27213 as an LPMO, it lacks a complete carbohydrate‐binding module (CBM), which is also a characteristic of LPMO proteins. However, PHEC27213 displayed a putative chitin‐binding domain (Chitin_bind_3) that corresponded to several residues of the CBM33 from AA10 LPMO. The chitin‐binding activity of PHEC27213 was confirmed by a binding assay, which also showed its ability to bind cellulose to a lesser extent. On the contrary, an enzymatic assay showed that PHEC27213 was active only against chitin, catalysing it into small chitooligosaccharides. Therefore, the PHEC27213 protein was renamed PxLPMO1 (*P. xanthii* LPMO1). Furthermore, MALDI‐TOF‐MS analysis of the enzymatic reaction products showed that the product masses were consistent with the cleavage of the primary chain by C1 oxidation, producing predominantly aldonic acid oligosaccharides, as has been previously described for AA10 and AA11 LPMOs (Hemsworth et al., 2014; Vaaje‐Kolstad et al., 2010).

The lytic activity of PxLPMO1 was more similar to AA11 LPMO than to AA10 LPMO, because AA10 LPMO‐released chitooligosaccharides showed even‐numbered degrees of polymerization (DP4, DP6, DP8, etc.), while in the case of AA11 LPMO, the chitooligosaccharides presented any degree of polymerization (DP5, DP6, DP7, DP8, etc.). However, in the case of PxLPMO1, the product masses predominantly showed a degree of polymerization of 5, which is different from that previously described for AA10 and AA11 LPMOs. Furthermore, the catalytic activity of PxLPMO1 is also different from EWCA effectors that randomly catalyse immunogenic chitooligosaccharides through endochitinase activity (Martínez‐Cruz et al., 2021). This new chitin catalyst pattern was analysed further by molecular docking. The docking results obtained with SwissDock were consistent with the results obtained by MALDI‐TOF‐MS analysis of the enzymatic reaction products, as the proposed chitooligosaccharide‐binding site is located very close to the histidine brace, placing the C1 of DP5 as the closest oxidation site. However, the proposed chitin‐binding site is different from those previously described in other LPMOs (Hemsworth et al., 2014). At this point, we must mention that our docking analysis has two important limitations. First, the protein model used is a computational prediction and not a crystallized protein. Second, the ligand used is a large molecule with too many atoms and it is too flexible, which are the main reasons why no results or favourable‐binding sites were obtained when using additional docking software, such as Glide or AutoDock Vina (data not shown). Even if docking algorithms are remarkably accurate these days, each docking program relies on the ability of the docking server to generate poses approximate to experimental poses. In other words, different programs rely on different algorithms when generating predictive simulations, which may explain the strong discrepancy when comparing results obtained with Glide and AutoDock Vina with those obtained with SwissDock. Therefore, our docking results, while consistent with enzymatic data, must be confirmed experimentally.

In our view, the recent discovery of LPMO activity (Vaaje‐Kolstad et al., 2010), the identification of new families in the last 2 years (Couturier et al., 2018; Filiatrault‐Chastel et al., 2019; Sabbadin et al., 2018), and the fact that these enzymes have not yet been described in pathogenic organisms suggest that PxLPMO1 could be a novel type of chitin LPMO specifically involved in pathogenic processes.

To validate this hypothesis, RNAi silencing of *PxLPMO1* was carried out by the previously described ATM‐HIGS method (Martínez‐Cruz et al., 2018). The results showed a notable reduction in *P. xanthii* growth that was even higher than the reduction caused by RNAi silencing of the melon *CmMLO1* gene, the positive control for RNAi‐induced resistance typically used in ATM‐HIGS experiments with *P. xanthii* (Martínez‐Cruz et al., 2018). Moreover, the accumulation of ROS by epidermal cells was also higher than that observed with the silencing of *CmMLO1*. The activation of the oxidative burst in the host when *PxLPMO1* was silenced, together with the fact that the infiltration of the product from the enzymatic reaction catalysed by PxLPMO1 using colloidal chitin as a substrate was not able to trigger the oxidative burst elicited by the substrate, indicated a role for PxLPMO1 in the suppression of plant defence response activation. For this reason, the melon *CmCERK1* gene was co‐silenced together with the *PxLPMO1* gene. CERK1 is a transmembrane kinase receptor indispensable for the perception of chitin oligosaccharides by the plant; because the absence of CERK1 causes the loss of the ability to respond to oligosaccharide elicitors, the production of ROS is suppressed, MAP kinases are not activated, and defence‐related genes are not expressed (Gimenez‐Ibanez et al., 2009; Lee et al., 2013; Miya et al., 2007; Petutschnig et al., 2014). The silencing of both genes caused a reversion to the wild‐type phenotype, that is, to the normal growth of *P. xanthii* and the suppression of the oxidative burst, indicating that CmCERK1 was involved in the PTI response mediated by chitin when *PxLPMO1* was silenced. Altogether, we can conclude that the mechanism of action of PxLPMO1 is to suppress the activation of plant immunity by avoiding the recognition of haustorial chitin by plant cells. This mechanism could be related to the dimerization of CERK1 induced by chitooligosaccharides. It has been reported that chitin octamers (DP8) induce CERK1 dimerization and thereby activate the plant defence mechanisms mediated by chitin, whereas DP5 and shorter oligosaccharides do not promote CERK1 dimerization and do not activate the subsequent signalling response. Moreover, it has been reported that DP5 attenuated the dimerization and, subsequently, the immune response caused by DP8 or longer chitooligosaccharides (Liu, Liu et al., 2012). Because PxLPMO1 catalyses chitin mostly into chitooligosaccharides with DP5, this enzyme activity should prevent the dimerization of CmCERK1.

**FIGURE 8:**
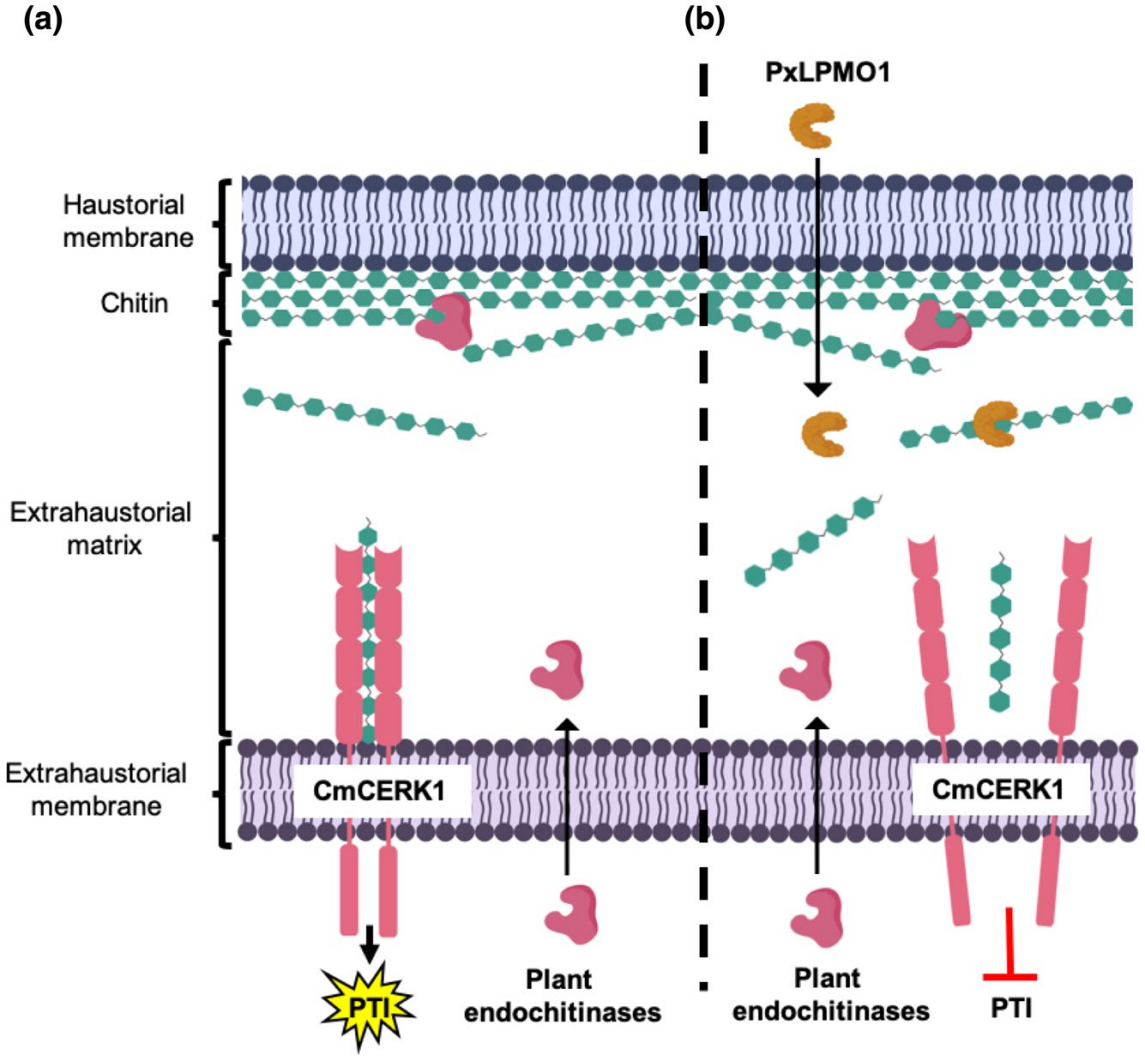
Schematic representation of the proposed role of PxLPMO1. (a) In the absence of PxLPMO1, plant‐secreted endochitinases release chitin fragments from the haustorial cell wall that can be recognized by the CmCERK1 receptor, activating chitin‐triggered immunity (PTI). (b) When PxLPMO1 is released into the extrahaustorial matrix, the enzyme catalyses the transformation of immunogenic chitin fragments into small chitooligosaccharides, predominantly DP5 oligosaccharides, that cannot induce CmCERK1 dimerization, thereby suppressing the activation of PTI. To reduce complexity, other components of the fungal cell wall have been omitted, such as β‐glucans and cell wall proteins

The strong expression of two endochitinases in melon plants infected with *P. xanthii* and the fact that they are coexpressed with *PxLPMO1* suggest that, after its putative secretion, PxLPMO1 may localize to the extrahaustorial matrix and act on large chitin fragments released from the haustorial cell wall by plant endochitinases, catalysing them into small oligosaccharides and thus avoiding chitin recognition by CmCERK1, thereby suppressing the activation of chitin‐triggered immunity (Figure 8). In this model, we anticipate that CmCERK1 should be located in the extrahaustorial membrane, as previously described for other plant receptors responsible for pathogen recognition, such as RPW8.2, which enhances hydrogen peroxide accumulation and callose encasement of the haustorial complex (Kim et al., 2014; Wang et al., 2009). Obviously, further experiments are necessary to confirm this prediction. To date, no mechanisms have been described to explain the suppression of PTI during the development of powdery mildew haustoria. Micali et al. (2011) did not find chitosan or any other modification in the haustorial chitin, and they suggested the presence of “unknown mechanisms” that allow for the suppression of chitin‐triggered immunity. To our knowledge, PxLPMO1 is the first mechanism described to avoid the activation of PTI induced by chitin in powdery mildew haustoria.

Although at first we only found orthologues of *PxLPMO1* in pathogenic fungi and, to a lesser extent, in endophytic and endomycorrhizal fungi, a deeper analysis using the specific proteomes of different haustorium‐forming fungal pathogens allowed us to detect PxLPMO1‐like proteins in other powdery mildew and rust fungi. These proteins share common features with other known chitin LPMOs, such as the histidine brace present in all LPMOs, the protein folding of AA11 LPMOs, or the presence of several residues of the CBM33 typical of the AA10 LPMOs. However, these proteins are phylogenetically separated from chitin LPMOs, suggesting that the LPMO‐like proteins from haustorium‐forming fungal pathogens are a putative new group of LPMO proteins that could be specifically related to the suppression of chitin recognition by the host. These LPMO‐like proteins present low sequence identities with PxLPMO1; however, they have the same structure, that is, they are small proteins with a signal peptide, they have the histidine brace, they have a similar fold, they have a putative chitin‐binding domain, and they have a highly variable C‐terminal domain. Although it is tempting to speculate that LPMO‐like proteins from haustorium‐forming fungal pathogens could act similar to PxLPMO1, the putative membrane localization and predicted GPI‐anchors of some of them differs from PxLPMO1, which suggests that they could carry out different functions according to their C‐terminal domains. This fact could explain the binding of chitin by the C‐terminal domain of PxLPMO1 that the LPMO‐like proteins located in the membrane could not perform. This is the case of other LPMO‐like proteins, such as the LPMO‐like proteins from the ectomycorrhizal fungus *Laccaria bicolor* (Labourel et al., 2020) or from the yeast pathogen *Cryptococcus neoformans* (Garcia‐Santamarina et al., 2020), both belonging to the X325 family, as well as the 6BIH protein from *L. arvalis*, which has a protein fold similar to PxLPMO1. These proteins are essential for symbiosis and pathogenesis, respectively, but they are GPI‐anchored proteins and carry out functions different from canonical LPMOs. In the case of *L. bicolor,* the protein acts as a chitin‐reorganization protein, but it is incapable of catalysing chitin, while the *C. neoformans* protein acts primarily as a copper acquisition protein and is incapable of catalysing cellulose (Garcia‐Santamarina et al., 2020; Labourel et al., 2020). However, these observations do not exclude the possibility that they exhibit enzyme activity on other yet unidentified polysaccharide substrates.

To conclude, our findings show the presence of a novel class of chitin LPMOs in *P. xanthii* with orthologues in different ascomycete ectomycorrhizal and fungal pathogens. We also suggest the existence of LPMO‐like proteins in haustorium‐forming fungal pathogens that can carry out different functions related to fungal development or pathogenesis, according to their C‐terminal domains. Regarding pathogenesis, we demonstrate the involvement of LPMO enzymes in the suppression of chitin signalling, which reinforces the idea that the evolution of molecular strategies to disarm the activation of chitin‐triggered immunity is mandatory for the successful colonization of plant environments by fungi, especially haustorium‐forming fungal pathogens.

## 4 EXPERIMENTAL PROCEDURES

### 4.1 Plants, fungi, bacteria, and culture conditions

Zucchini (*Cucurbita pepo*) cv. Negro Belleza (Semillas Fitó) and melon (*Cucumis melo*) cv. Rochet (Semillas Fitó) plants were used for *P. xanthii* growth and RNAi silencing experiments, respectively. Plants were cultivated in growth chambers at 24 °C under a 16 hr light/8 hr dark cycle. For the growth of the *P*. *xanthii* isolate 2086, disinfected cotyledons of zucchini maintained in 8‐cm Petri dishes with Bertrand medium were used as previously described (Álvarez & Torés, 1997). For agroinfiltration and RNAi silencing, *Agrobacterium tumefaciens* C58C1 was used and grown at 28 °C in Luria Bertani (LB) medium with rifampicin (50 μg/ml) and spectinomycin (100 μg/ml) when required. For the maintenance, construction, and propagation of RNAi silencing vectors, *E. coli* strains DH5α or DB3.1 were used, whereas for protein expression, *E. coli* Bl21‐CodonPlus‐RIL was used. *E. coli* strains were grown at 37 °C in LB medium with ampicillin (100 μg/ml), spectinomycin (100 μg/ml), or kanamycin (50 μg/ml) when required.

### 4.2 Sequence analysis, protein modelling, and molecular docking

The unigene PHEC27213 was selected from the previously published *P. xanthii* secretome (Polonio, Seoane, et al., 2019). This unigene was the most expressed gene among the expressed haustorium‐specific proteins; however, PHEC27213 had no annotated function. To analyse the signal peptide and select the mature protein sequence, the SignalP v. 4.1 server (Petersen et al., 2011) was used. To elucidate the putative function of PHEC27213, the mature protein sequence of PHEC27213 was employed to construct 3D models using the I‐TASSER (Zhang, 2008), Phyre2 (Kelly et al., 2015), and IntFOLD (McGuffin et al., 2019; Roche et al., 2011) servers. The identification of domains in the mature protein was carried out by the Pfam (Sonnhammer et al., 1998), dbCAN2 (Zhang et al., 2018), and MotifScan (Pagni et al., 2007) servers, whereas the UniProt server (Apweiler et al., 2004) was used to perform protein alignments with several AA10 LPMO sequences retrieved from Protein Data Bank (PDB).

To identify the putative site of chitin binding to the 3D predicted model of PHEC27213, the chitin heptamer molecule (DP7) was taken from the PDB model 5GQB, a Lepidoptera‐exclusive insect chitinase from *Ostrinia furnacalis* (Liu et al., 2017), and used to perform an automated molecular docking analysis using the SwissDock server (www.swiss‐dock.ch/docking) (Grosdidier et al., 2011). The docking was performed using the “Accurate” parameter with default parameters otherwise and no region of interest defined (blind docking). Modelling and docking results were visualized using UCSF Chimera software (Pettersen et al., 2004).

To investigate the presence of putative orthologues of PxLPMO1 in other fungi, BLASTp (BLAST+ v. 2.7.1) against the NCBI‐nr databases was used (*E* value < 1E−5); to investigate the presence of LPMO‐like proteins in other haustorium‐forming fungal pathogens, BLASTp (BLAST+ v. 2.7.1; *E* value < 1E−5) was also used against the available proteomes of *Blumeria graminis* f. sp. *hordei* (UNSH00000000) (Frantzeskakis et al., 2018), *Erysiphe necator* (JNVN00000000) (Jones et al., 2014), *Erysiphe pulchra* (PEDP00000000) (Wadl et al., 2019), *Golovinomyces cichoracearum* (MCBR00000000) (Wu et al., 2018), *Puccinia graminis* f. sp. *tritici* (AAWC01000000) (Duplessis et al., 2011), *Puccinia triticina* (ADAS00000000), *Puccinia striiformis* f. sp. *tritici* (AJIL00000000) (Cuomo et al., 2017), *Puccinia sorghi* (LAVV00000000) (Rochi et al., 2018), and *Melampsora larici‐populina* (AECX00000000) (Duplessis et al., 2011). Similarly, to investigate the presence of paralogs and LPMO‐like proteins in the *P. xanthii* genome, the two recently assembled genomes, JACSEY000000000 (Polonio et al., 2021) and JAAAXZ000000000 (Kim et al., 2021), were analysed by BLASTNn and tBLASTn searches (BLAST+ v. 2.7.1; *E* value < 1E−5), respectively.

To delve into the sequences and characteristics of these putative orthologues, a set of tools were used: SignalP v. 4.1 (to predict the signal peptide) (Petersen et al., 2011), Clustal W‐OMEGA (to perform phylogenetic analysis) (Sievers and Higgins, 2014), I‐TASSER (to perform 3D model prediction) (Zhang, 2008), DeepLoc (to predict the exact protein locations) (Almagro Armenteros et al., 2017), PredGPI (to predict putative GPI‐anchor domains) (Pierleoni et al., 2008), UniProt (to carry out the protein alignments) (Apweiler et al., 2004), and MEGA X (to generate the phylogenetic tree) (Kumar et al., 2018).

To identify melon leaf chitinases differentially expressed in response to *P. xanthii* infection, a previous RNA‐Seq analysis of the early stages of melon powdery mildew disease was used (Polonio, Pineda, et al., 2019). In this study, the raw reads from the melon plants infected with *P. xanthii* were trimmed and aligned to the melon reference transcriptome to perform an expression analysis. Uniquely localized reads were used to calculate those differentially expressed genes between the control and infected plants. A *p* value <.05 and log_2_(fold change) >1 were considered the significance threshold for each gene. Two differentially expressed plant endochitinases, an acidic endochitinase (XM_008439336.2) and an EP3‐like endochitinase (XM_008446389.2), were selected and used for expression analysis in conjunction with the PHEC27213 unigene.

### 4.3 DNA and RNA isolation and cDNA synthesis

To isolate DNA and RNA from *P. xanthii*‐infected zucchini cotyledons, the cotyledons were frozen in liquid nitrogen and ground with a mortar and pestle. Genomic DNA was isolated using the MasterPure Yeast DNA Purification Kit (Epicentre), and total RNA was extracted using the TRI reagent (Sigma‐Aldrich), following the manufacturers’ instructions. Total RNA was quantified using a NanoDrop 2000 spectrophotometer (Thermo Fisher Scientific), and cDNA synthesis was performed using random primers (Thermo Fisher Scientific) and Superscript III reverse transcriptase (Thermo Fisher Scientific) according to the manufacturer's recommendations.

### 4.4 Construction of protein expression and RNAi silencing vectors

The plasmids used in this work are listed in Table S1 and are schematically represented in Figure S6. For the in vitro expression of the PHEC27213 protein, the complete open reading frame sequence, without the nucleotides corresponding to the signal peptide and the stop codon, was amplified using the specific primers listed in Table S2, which contained *Nde*I and *Xho*I restriction sites. Subsequently, the *PHEC27213* amplicon was digested with fast digest restriction enzymes (Thermo Fisher Scientific) and cloned into the pET30b plasmid using T4 ligase (Thermo Fisher Scientific) according to the manufacturer's recommendations. The pET30b plasmid places a 6 × His‐tag at both the N‐terminus and the C‐terminus of the protein, but with *Nde*I and *Xho*I combined digestion, only the 6 × His‐tag at the C‐terminus was conserved. The resulting expression vector, pPHEC27213‐EXPR, was propagated and maintained in *E. coli* DH5α and verified by PCR amplification, digestion, and sequencing. Finally, for the expression of the protein, pPHEC27213‐EXPR was introduced into *E. coli* Bl21‐CodonPlus‐RIL by electroporation.

For RNAi silencing of the *PxLPMO1* (*PHEC27213*) gene, the vector pB7GWIWG2(II) (Karimi et al., 2002) and Gateway cloning technology (Invitrogen) were used as previously described (Martínez‐Cruz et al., 2018). Specific primers with attB1 or attB2 tails (Table S2) were used to amplify a 315‐bp fragment of *PxLPMO1* from the previously obtained cDNA (see above). The resulting plasmid, pPxLPMO1‐RNAi, was checked by PCR and sequencing. For RNAi silencing experiments, the pCmMLO1‐RNAi plasmid, containing a 412‐bp fragment of the melon *CmMLO1* gene, which encodes a plant transmembrane protein involved in abiotic and biotic stresses and whose loss‐of‐function mutation protects plants from powdery mildew infection (Kusch & Panstruga, 2017), was used as a positive control (Martínez‐Cruz et al., 2018), and the empty pB7GWIWG2(II) vector was used as a negative control (Table S1). In addition, for RNAi silencing of the melon chitin receptor kinase gene *CmCERK1*, the plasmid pCmCERK1‐RNAi (Table S1) containing a 614‐bp fragment of the melon *CmCERK1* gene was used (Martínez‐Cruz et al., 2021). All of these plasmids were propagated and maintained in *E. coli* strains DH5α or DB3.1. For the RNAi silencing experiments, these plasmids were introduced into *A. tumefaciens* C58C1 by electroporation.

### 4.5 Protein expression and purification

For in vitro production of the PHEC27213 protein, *E. coli* BL21‐CodonPlus‐RIL harbouring the pPHEC27213‐EXPR expression vector was used. For this purpose, *E. coli* cells were grown in LB medium with kanamycin (50 μg/ml) at 37 °C and induced with 0.5 mM IPTG (isopropyl‐β‐d‐thiogalactopyranoside) when they reached an OD_600 nm_ of 0.4. Subsequently, the cells were incubated overnight at 16 °C in an orbital shaker at 80 rpm. After incubation, *E. coli* cells were harvested by centrifugation at 8,000 × g for 5 min at 4 °C and the resulting pellet was stored at −80 °C overnight to increase the yield from the protein extraction. The purification of soluble PHEC27213 protein, which included a 6 × His‐tag at the C‐terminus, was carried out using a Protino Ni‐TED 2000 Packed Columns (Macherey‐Nagel GmbH & Co. KG) according to the manufacturer's instructions. The purified recombinant proteins were then desalted using Sephadex G‐25 in PD‐10 desalting columns (GE Healthcare) and 0.1 M sodium phosphate buffer, pH 7. Protein purification was confirmed using Mini‐PROTEAN Stain‐Free Precast Gels (Bio‐Rad) and visualized in a ChemiDoc XRS+ System (Bio‐Rad). Finally, the protein concentration was estimated by the Protein Concentration Calculator webserver (https://www.aatbio.com/tools/calculate‐protein‐concentration) using the absorbance value at 280 nm, the extinction coefficient, and the molecular weight of the protein calculated from the Expasy webserver (https://www.expasy.org/).

### 4.6 Binding activity assays

Prior to binding assays, cellulose, and colloidal chitin solutions were prepared. In the case of cellulose, Avicel PH‐101 crystalline cellulose was used. In the case of chitin, a suspension of colloidal chitin was prepared as described by Souza et al. (2009). Briefly, 5 g of chitin powder from shrimp shells (Sigma‐Aldrich) was added to 60 ml of a solution of concentrated HCl and incubated with stirring overnight. This mixture was then added to 200 ml of previously cooled 95% ethanol and incubated overnight with stirring again. The precipitate was centrifuged at 4 °C for 20 min at 5,000 × g and then filtered using filter paper. The resulting colloidal chitin was washed several times until reached a pH of 7 and was then stored at 4 °C in dark.

To perform binding assays, 250 μl of 1% solutions of colloidal chitin or cellulose were centrifuged at 13,000 × g for 5 min at 4 °C. The resulting pellet was resuspended in 250 μl of 1 mg/ml purified PHEC27213 protein in 0.1 M sodium phosphate buffer (pH 7) or bovine serum albumen (BSA) (negative control). The mixtures were incubated at 4 °C for 1 hr with gentle manual agitation every 15 min. Later, the mixtures were centrifuged at 13,000 × g for 5 min at 4 °C. After centrifugation, the supernatants and pellets were separated, and the proteins present in the supernatant were visualized using Mini‐PROTEAN Stain‐Free Precast Gels (Bio‐Rad) in a ChemiDoc XRS + system (Bio‐Rad) and quantified as described above as an indicator of the proteins unbound to colloidal chitin or cellulose.

### 4.7 LPMO activity assay

To validate the LPMO activity predicted for the PHEC27213 protein, chitin, and cellulose degradation assays were performed (Hemsworth et al., 2014). In these assays, 1 μM purified PHEC27213 was added to a reaction mixture containing 2 mg/ml colloidal chitin, hepta‐*N*‐acetylchitoheptaose (DP7, *m*/*z* = 1,440.36) or cellulose, and 1 mM ascorbic acid as a reducing agent in 0.1 M sodium phosphate buffer (pH 7). The reactions were incubated overnight at 37 °C with shaking at 800 rpm in a thermomixer (Eppendorf). After incubation, the samples were centrifuged at 8,000 × g for 5 min at 4 °C. The oligosaccharides resulting from the enzymatic reactions were analysed by MALDI‐TOF‐MS using an Ultraflex MALDI‐TOF/TOF instrument (Bruker Daltonics GmbH) with a nitrogen 337 nm laser beam as described by Vaaje‐Kolstad et al. (2010). Penta‐*N*‐acetylchitopentaose (DP5, *m*/*z* = 1,033.98) was used as control of native unoxidized chitooligosaccharide. Oligosaccharides were detected according to the product masses of the resulting peaks previously described by Vaaje‐Kolstad et al. (2010) and Hemsworth et al. (2015).

### 4.8 RT‐qPCR and qPCR

The expression analysis of *P*. *xanthii* and melon genes and the molecular estimation of *P. xanthii* biomass were carried out by RT‐qPCR and qPCR, respectively. The primers used for these analyses (Table S2) were designed using Primer3 software (Koressaar & Remm, 2007; Thornton & Basu, 2011). For gene expression analysis, total RNA was extracted and used to synthetize cDNA as described above. As normalization reference genes, the *P. xanthii* translation elongation factor 1‐alpha gene *PxEF1* (MK249653) and the *C*. *melo* actin‐7 gene *CmACT7* (XM_008462689.2) were used (Polonio, Pineda, et al., 2019; Polonio, Seoane, et al., 2019). For the molecular estimation of fungal biomass, total DNA was isolated from agroinfiltrated and infected melon cotyledons as described above. For this purpose, the *P. xanthii* β‐tubulin gene *PxTUB2* (KC333362) and the *C. melo* actin‐7 gene *CmACT7* (XM_008462689.2) were quantified and the *P*. *xanthii*/*C. melo* genomic DNA ratio was calculated as previously described (Vela‐Corcía et al., 2016). RT‐qPCR and qPCR assays were carried out in a CFX384 Touch Real‐Time PCR detection system (Bio‐Rad) using SsoFast EvaGreen Supermix (Bio‐Rad) according to the manufacturer's indications with the following conditions: enzyme activation step at 95 °C for 30 s, followed by 40 cycles at 95 °C for 5 s and 65 °C for 5 s. After amplification, the data were processed by CFX Manager Software (Bio‐Rad), and the amplicon sizes were confirmed by visualization on 2% agarose gels.

### 4.9 *Agrobacterium tumefaciens*‐mediated host‐induced gene silencing (ATM‐HIGS) assay

To study the role of *PxLPMO1* in *P. xanthii* development, an ATM‐HIGS assay was carried out as previously described (Martínez‐Cruz et al., 2018). The RNAi silencing plasmids pPxLPMO1‐RNAi and CmCERK1‐RNAi, as well as the RNAi‐positive control plasmid pCmMLO1‐RNAi and the pB7GWIWG2(II) empty vector were introduced into *A. tumefaciens* C58C1 by electroporation. Later, transformed *A. tumefaciens* cells were induced with 200 μM acetosyringone and grown in 5 ml LB medium supplemented with rifampicin (50 μg/ml) and spectinomycin (100 μg/ml) or kanamycin (50 μg/ml) at 28 °C and 200 rpm in an orbital shaker overnight. Subsequently, the different *Agrobacterium* cultures corresponding to the different RNAi silencing constructs were washed twice in washing buffer and 200 μM acetosyringone. To induce the Vir proteins, the bacterial cells were incubated for 2 hr at room temperature in the same washing buffer without agitation. Then, the *Agrobacterium* cells were centrifuged for 10 min at 1,800 × g and 28 °C and resuspended in MES buffer, and then their OD_600 nm_ was adjusted to 0.5–1.0 in MES buffer. Finally, 1‐ml syringes without the needle were used to perform the agroinfiltrations into the abaxial surface of melon cotyledons with the different cell suspensions. For the co‐silencing experiments, equal volumes of the *Agrobacterium* cell suspensions carrying the pPxLPMO1‐RNAi and pCmCERK1‐RNAi silencing constructs were mixed before agroinfiltration. The agroinfiltrated cotyledons were maintained in a growth chamber under a 16 hr light/8 hr dark cycle at 24 °C for 24 hr until inoculation with fresh *P. xanthii* conidial suspensions (10^5^ conidia/ml) by pulverization. Then, the cotyledons were maintained under the same conditions until analysis.

### 4.10 Haustorial counts and visualization of fungal development and oxidative bursts

For the quantification of the number of haustoria after RNAi silencing, the visualization of fungal development and to analyse the activation of oxidative burst (e.g., the accumulation of H_2_O_2_), the 3,3′‐diaminobenzidine (DAB) method (Thordal‐Christensen et al., 1997) proposed by Martínez‐Cruz et al. (2018) was performed. Briefly, discs of 1 cm in diameter were taken from agroinfiltrated and *P. xanthii*‐infected cotyledons and incubated in 1 mg/ml DAB (pH 3.8) overnight in the dark and at room temperature. After incubation, the discs were decoloured in boiling ethanol and observed by light microscopy using an Eclipse E800 microscope (Nikon). With these preparations, the haustoria can be visualized as black spots, whereas the hyphae are stained brown. In the same preparations, epidermal cells with brown‐red precipitates are reactive cells with H_2_O_2_ accumulation.

### 4.11 Statistical analysis

When required, statistical analysis of data was carried out by IBM SPSS v. 20 software (SPSS) using Pearson's correlation coefficient or Fisher's least significant difference test (LSD).

## CONFLICT OF INTEREST

Authors declare no competing financial interest.

## Supporting information

**TABLE S1** Plasmids used in this study

## Data Availability

The sequence of *PxLPMO1* can be found in the GenBank database at https://www.ncbi.nlm.nih.gov/genbank/ with the accession no. MT234390.
