# Supplementary figures and images for: A haustorial‐expressed lytic polysaccharide monooxygenase from the cucurbit powdery mildew pathogen Podosphaera xanthii contributes to the suppression of chitin‐triggered immunity
Source: Mol Plant Pathol. 2021 Mar 19;22(5):580–601. doi: 10.1111/mpp.13045 (PMC8035642; doi:10.1111/mpp.13045)

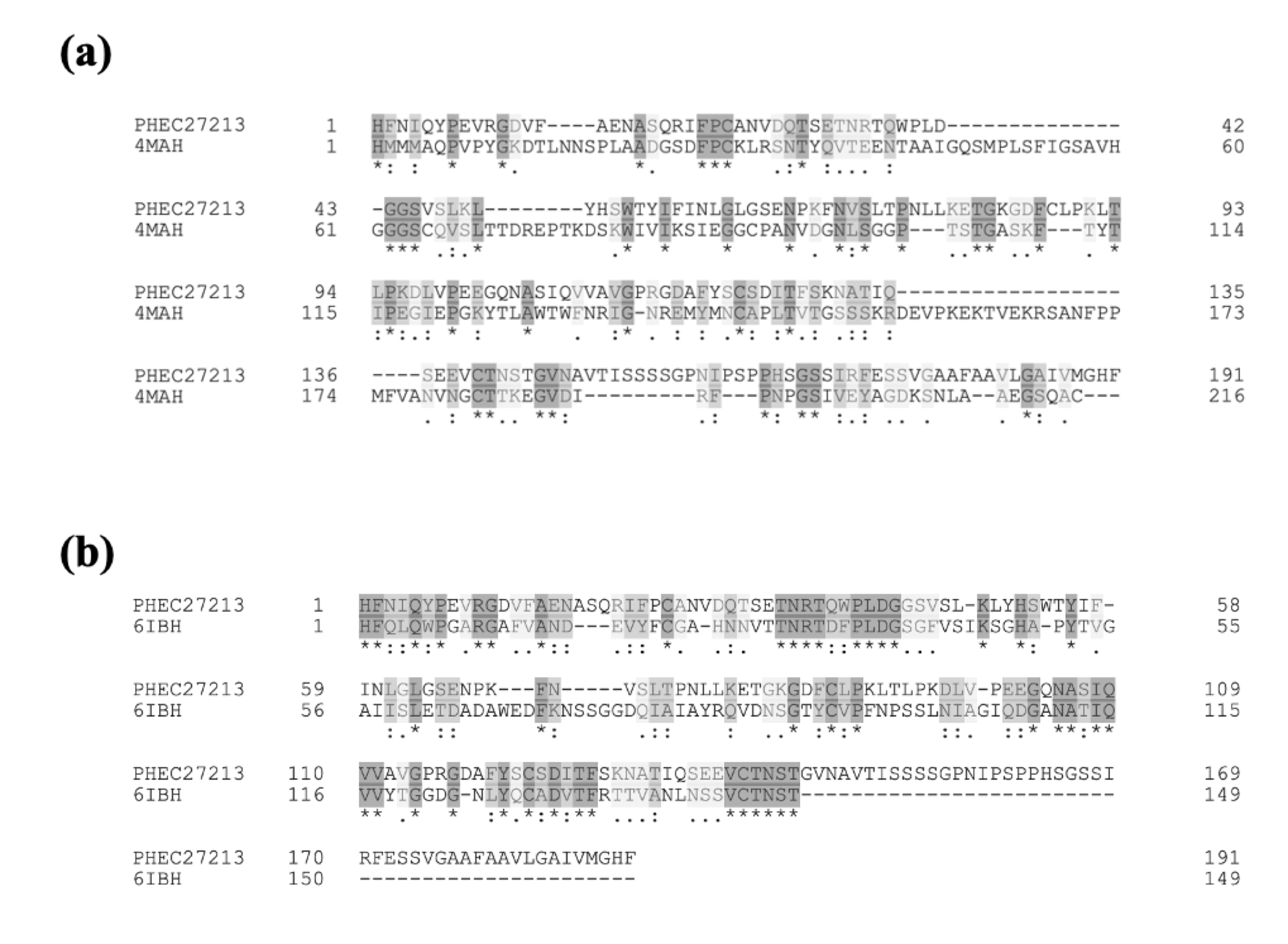

Supplement: Supplementary file 1 [file MPP-22-580-s004.tiff]

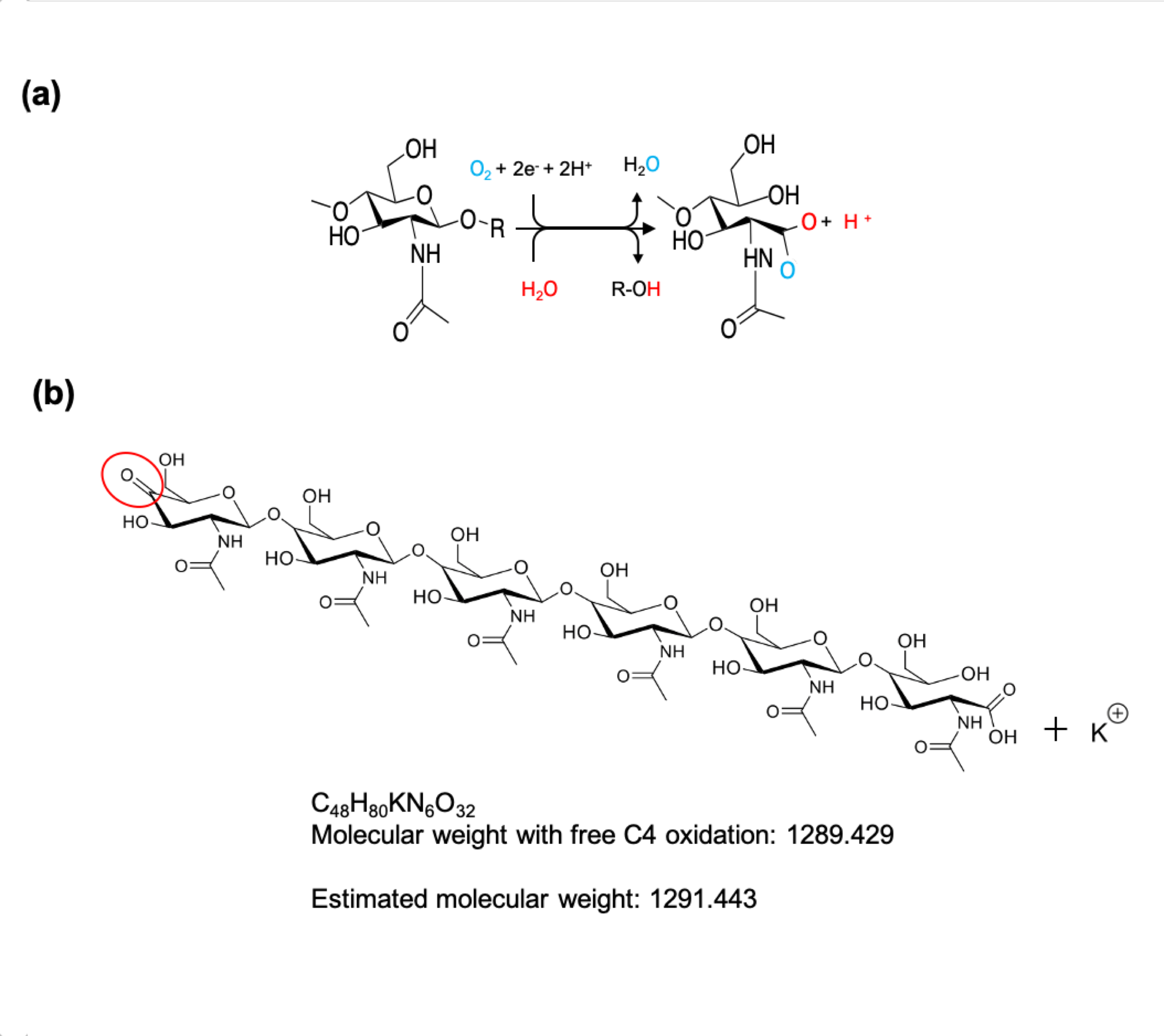

Supplement: Supplementary file 2 [file MPP-22-580-s002.tiff]

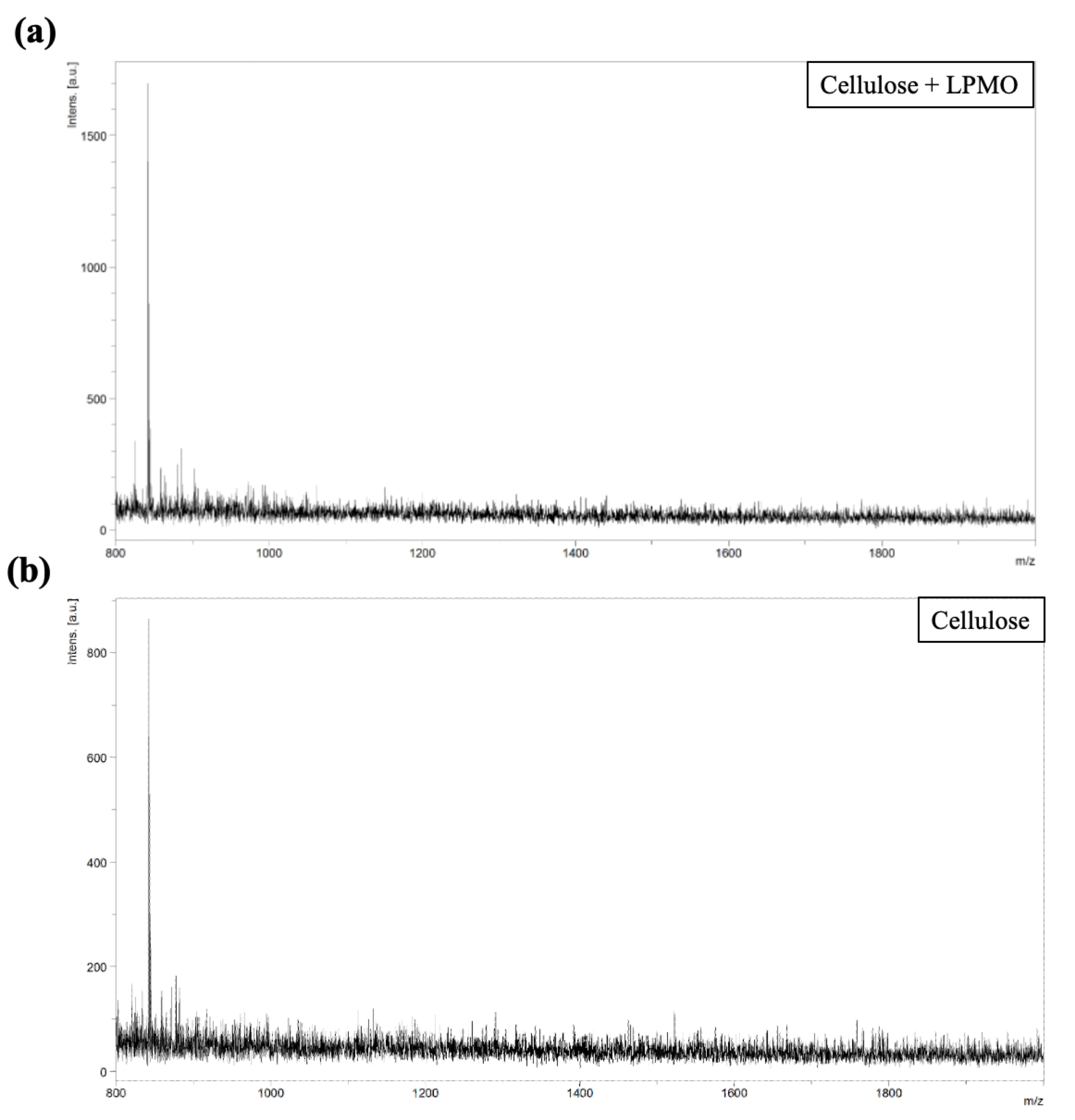

Supplement: Supplementary file 3 [file MPP-22-580-s007.tiff]

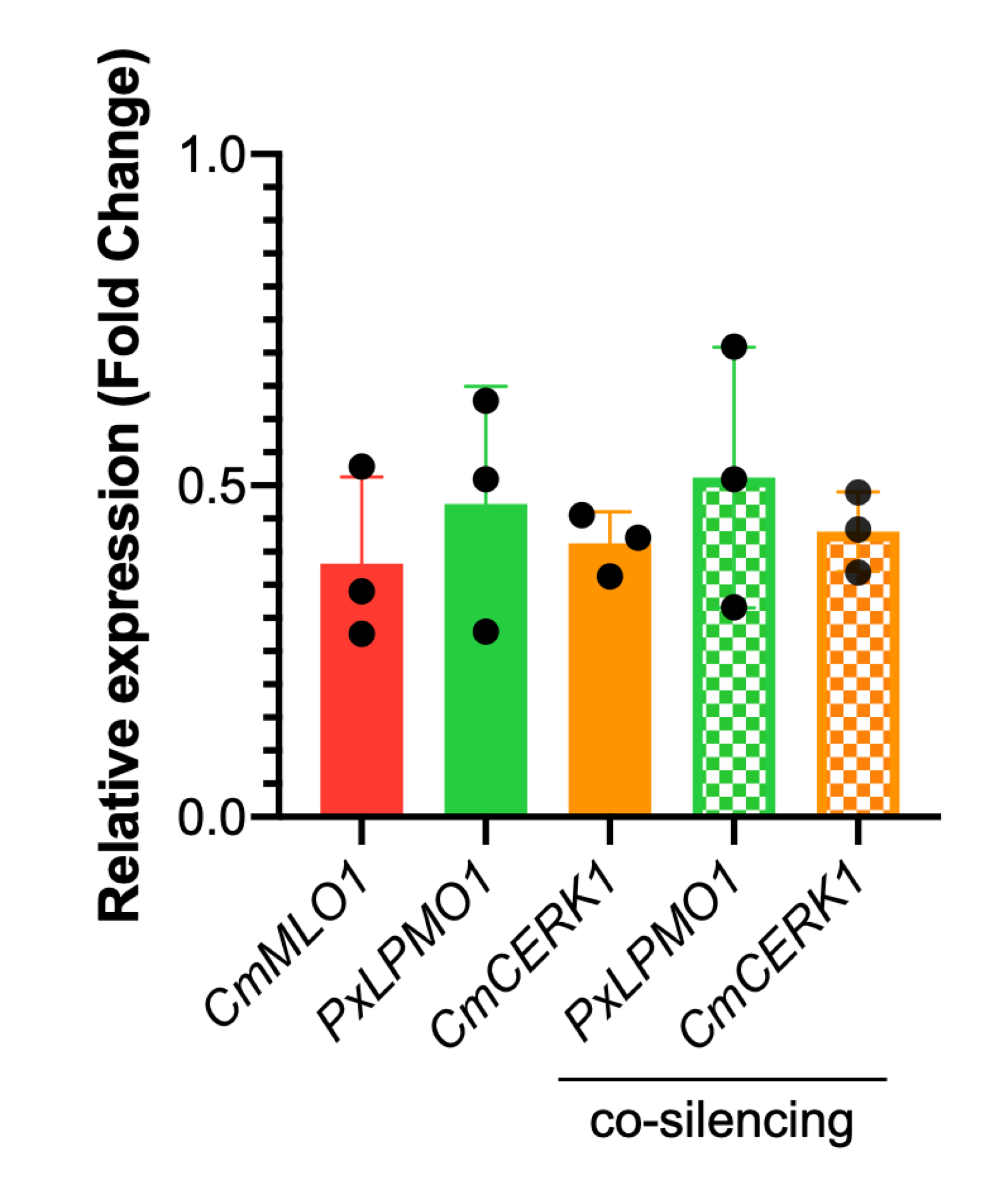

Supplement: Supplementary file 4 [file MPP-22-580-s001.tiff]

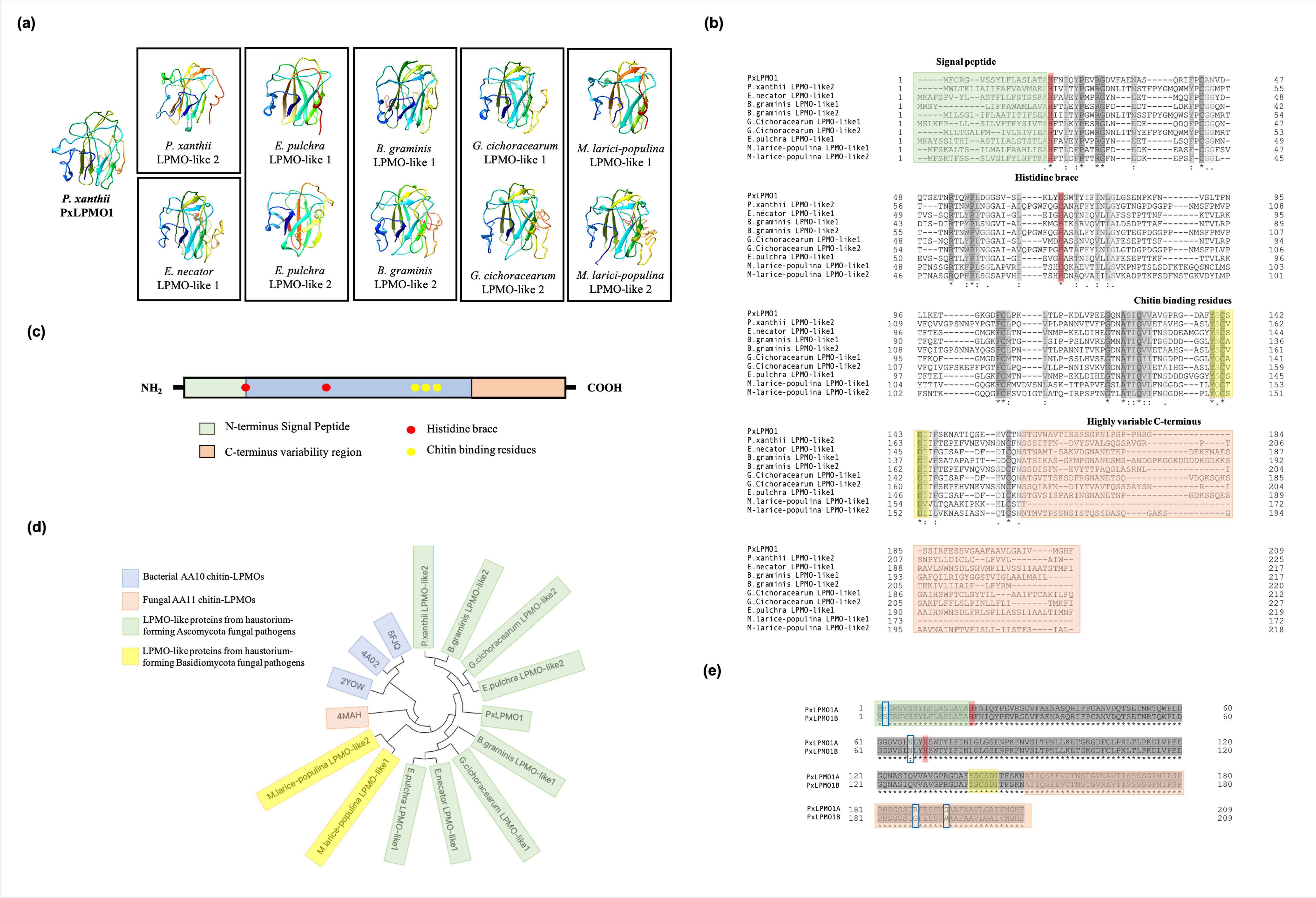

Supplement: Supplementary file 5 [file MPP-22-580-s003.tiff]

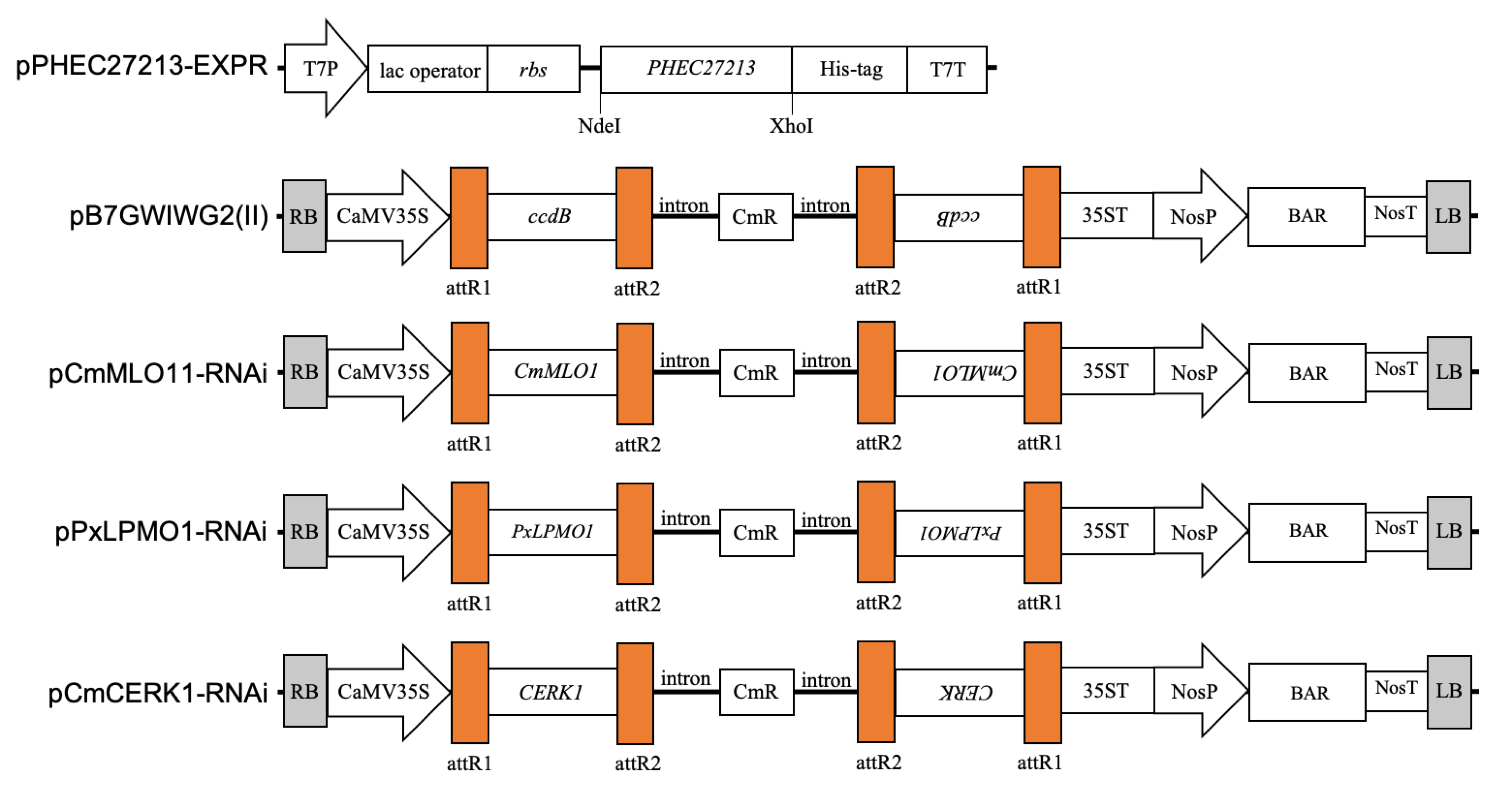

Supplement: Supplementary file 6 [file MPP-22-580-s006.tiff]
