# Supplementary material for: A haustorial‐expressed lytic polysaccharide monooxygenase from the cucurbit powdery mildew pathogen Podosphaera xanthii contributes to the suppression of chitin‐triggered immunity
Source: Mol Plant Pathol. 2021 Mar 19;22(5):580–601. doi: 10.1111/mpp.13045 (PMC8035642; doi:10.1111/mpp.13045)
Supplement: Supplementary file 7 — TABLE S1 Plasmids used in this study [file MPP-22-580-s008.docx]

**Table S1.** Plasmids used in this study.

| **Plasmid^a^** | **Features^b^** | **Reference** |
| --- | --- | --- |
| pB7GWIWG2(II) | Empty vector. Negative control for gene silencing experiments. | Karimi *et al.* 2002 |
| pCmMLO1-RNAi | Positive control for gene silencing experiments. Silencing vector containing an RNA hairpin with a 412 bp fragment of melon *CmMlo1* gene. | Martínez-Cruz *et al.* 2018 |
| pCERK1-RNAi | Silencing vector containing an RNA hairpin with a 614 pb fragment of melon *CERK1* gene | Martínez-Cruz *et al*. 2021 |
| pPxLPMO1-RNAi | Silencing vector containing an RNA hairpin with a 360 bp fragment of *PxHSLPMO1*. | This study |
| pPHEC27213-EXPR | Expression vector carrying the complete ORF of *PHEC27213* without SP and codon stop. | This study |

**^a^**RNAi, RNA interference.

**^b^**SP, secretion signal peptide.
