# Supplementary material for: A haustorial‐expressed lytic polysaccharide monooxygenase from the cucurbit powdery mildew pathogen Podosphaera xanthii contributes to the suppression of chitin‐triggered immunity
Source: Mol Plant Pathol. 2021 Mar 19;22(5):580–601. doi: 10.1111/mpp.13045 (PMC8035642; doi:10.1111/mpp.13045)
Supplement: Supplementary file 8 [file MPP-22-580-s005.docx]

**Table S2**. Primers used in this study.

| **Primer name** | **Sequence^a,b^** |
| --- | --- |
| **Plasmid construction** | |
| PxLPMO1si-F | 5’-AAAAAGCAGGCTCTGCGATGTATTCGCGGAGAAC -3’ |
| PxLPMO1si-R | 5’- AGAAAGCTGGGTGCTCGGTCCAACCGCAACAAC -3’ |
| NdeI-27213exp-F | 5’-*CATATG*CACTTTAATATTCAGTATCC -3’ |
| XhoI-27213exp nonSTOP-R | 5’- *CTCGAG*AAAGTGTCCCATAACAAT -3’ |
| attb1 | 5’-GGGGACAAGTTTGTACAAAAAAGCAGGCT-3’ |
| attb2 | 5’-GGGGACCACTTTGTACAAGAAAGCTGGGT-3’ |
| **Gene expression analysis** | |
| PxLPMO1q-F | 5’-AGTGTGCACCAATTCCACTG-3’ |
| PxLPMO1q-R  Endochitinase EP-3 like-F  Endoquitinase EP3-like-R  Acidic endochitinase-F  Acidic endochitinase-R | 5’- TCCCATAACAATTGCACCAA -3’  5’-TCCCCCACTTACTCCAAGTG-3’  5’- CGTCAACCGTTCCAATTCTT-3’  5’- AATCATCACAATCCTATCCAT-3’  5’- GAGGTTGAGGACCGGAGTTC -3’ |
| **Molecular estimation of fungal growth** | |
| Tubg-F | 5’-TTGTAGGAATCACATCCCTTTCTC-3’ |
| Tubg-R | 5’-TTCTTCCGGTTGCATGGGTGGTTC-3’ |
| Acting-F | 5’-GGCTGGATTTGCCGGTGATGATGC-3’ |
| Acting-R | 5’-GGAAGGAGGAAATCAGTGTGAACC-3’ |

**^a^** Underlined sequences correspond to the attb1 or attb2 primer adapter.

^b^ Italic and underlined sequences indicate the NdeI and XhoI restriction sites.
